# Glutamatergic and N-Acetylaspartate Metabolites in Bipolar Disorder: A Systematic Review and Meta-Analysis of Proton Magnetic Resonance Spectroscopy Studies

**DOI:** 10.3390/ijms23168974

**Published:** 2022-08-11

**Authors:** Jonathan Chabert, Etienne Allauze, Bruno Pereira, Carine Chassain, Ingrid De Chazeron, Jean-Yves Rotgé, Philippe Fossati, Pierre-Michel Llorca, Ludovic Samalin

**Affiliations:** 1 Service de Psychiatrie Adulte, CHU Clermont-Ferrand, CNRS, Institut Pascal, Université Clermont Auvergne, 58 Rue Montalembert, 63003 Clermont-Ferrand, France; 2 Biostatistics Unit (DRCI), CHU Clermont-Ferrand, Université Clermont Auvergne, 7 Place Henri Dunant, 63000 Clermont-Ferrand, France; 3 Imaging Department, CHU Clermont-Ferrand, CNRS, Institut Pascal, Université Clermont Auvergne, Clermont Auvergne INP, 58 Rue Montalembert, 63003 Clermont-Ferrand, France; 4 Service de Psychiatrie Adulte, Pitié-Salpêtrière Hospital, CNRS UMR 7593, 47-83 Bd de l’Hôpital, 75651 Paris, France

**Keywords:** NAA, N-acetylaspartate, glutamate, magnetic resonance spectroscopy, bipolar disorder, bipolar depression

## Abstract

The exact neurobiological mechanisms of bipolar disorder (BD) remain unknown. However, some neurometabolites could be implicated, including Glutamate (Glu), Glutamine (Gln), Glx, and N-acetylaspartate (NAA). Proton Magnetic Resonance Spectroscopy (^1^H-MRS) allows one to quantify these metabolites in the human brain. Thus, we conducted a systematic review and meta-analysis of the literature to compare their levels between BD patients and healthy controls (HC). The main inclusion criteria for inclusion were ^1^H-MRS studies comparing levels of Glu, Gln, Glx, and NAA in the prefrontal cortex (PFC), anterior cingulate cortex (ACC), and hippocampi between patients with BD in clinical remission or a major depressive episode and HC. Thirty-three studies were included. NAA levels were significantly lower in the left white matter PFC (wmPFC) of depressive and remitted BD patients compared to controls and were also significantly higher in the left dorsolateral PFC (dlPFC) of depressive BD patients compared to HC. Gln levels were significantly higher in the ACC of remitted BD patients compared to in HC. The decreased levels of NAA of BD patients may be related to the alterations in neuroplasticity and synaptic plasticity found in BD patients and may explain the deep white matter hyperintensities frequently observed via magnetic resonance imagery.

## 1. Introduction

Bipolar disorder (BD) is a mental illness with a lifetime prevalence of approximately 2.4% in the general population [1]. BD is characterized by successive mood episodes (depressive, manic/hypomanic, or mixed episodes) with inter-episodic periods, during which the patients are in clinical remission. This classical view, however, has been challenged as a large number of euthymic BD patients suffer numerous, persistent symptoms and/or cognitive problems during these periods of apparent clinical stability.

It is relatively common for BD to begin with depressive episodes since, on average, a patient with BD will have 2.5 major depressive episodes compared to 1 manic or hypomanic episode [2]. Consequently, the early diagnosis of BD may be very difficult and, thus, delayed from the onset of illness, which may result in inappropriate therapeutic management. In order to improve the management of BD patients, a better understanding of the mechanisms underlying BD remains of interest.

Magnetic resonance imaging (MRI) data have been used to identify several areas with altered structure or function in BD patients. The prefrontal regions tend to be hypo-activated, which alters the regulation of the hyperactive limbic regions and, therefore, leads to an increased emotional response [3,4]. This cortico-limbic dysregulation could be related to connectivity problems between the prefrontal and limbic regions [5]. This hypothesis seems to be supported by several arguments, notably that a decrease in total white matter volume was observed in BD patients [6] and that T2 or FLAIR hyperintensities were found in the deep prefrontal and periventricular white matter in various studies [7,8]. The emergence of diffusion MRI has supported the dysconnectivity model in BD. In a recent mega-analysis, BD patients showed significant damage to the corpus callosum and the cingulate, as well as to many other regions, including those allowing the association between the prefrontal and limbic regions [9]. Hyperactivity of the limbic regions in these patients, however, does not seem to be explained solely by poor frontal regulation, as volumetric anomalies were identified in anatomical MRI. For instance, Ellison-Wright et al. found a decrease in the volume of the rostral part of the anterior cingulate cortex (ACC) in BD patients when compared to healthy subjects [10]; a similar decrease was also observed in the meta-analysis of Bora et al., which similarly identified a decrease in the volume of the fronto-insular region in BD patients [11]. 

Several abnormalities have also been observed at the cellular level in BD. The hypothesis of dysfunction in the cerebral mitochondria has been the subject of numerous publications, with Morris et al. even proposing a model in which the various periods of mood episodes relate to mitochondrial energy production. Under this framework, manic episodes would be caused by an increase in energy production, whereas depressive episodes would be caused by a decrease in production [12]. Genetic studies have also highlighted involvement of the mitochondria in the pathophysiology of BD in such a way that mutations in mitochondrial DNA could be the origin of impairments in intracellular calcium signaling systems [13]. N-acetylaspartate (NAA) is the second most abundant molecule in the brain (after water), and its concentration is quantifiable by proton magnetic resonance spectroscopy (^1^H-MRS). NAA levels can be considered a marker of the integrity of mitochondrial energy metabolism, as they closely correlate with the concentration of ATP produced in the mitochondria [14,15,16]. The involvement of NAA in the pathophysiology of BD has been explored by many authors, including further evaluation in a meta-analysis of ^1^H-MRS studies, which found significantly lower levels of NAA in the basal ganglia and hippocampi of BD patients [17]. However, due to a lack of data, Kraguljac et al. could not analyze the levels of NAA according to the mood states of the patients. The euthymic and depressive phases seem to be the most interesting states because no alterations in NAA levels were identified in major depressive disorder (MDD) patients. Therefore, modifications during the depressive or euthymic phases of BD may help in the differential diagnostic process [18]. Furthermore, it is difficult to perform ^1^H-MRS studies during manic or hypomanic episodes without administering sedative treatments, and the impacts of these treatments on ^1^H-MRS data have not yet been fully studied.

The glutamatergic system plays important roles in various functions, including brain plasticity, neurotransmission, and energy metabolism [19]. The glutamatergic system is directly linked to NAA through the tricarboxylic acid cycle (TCA cycle), with the synthesis of NAA requiring transamination of glutamate (Glu) to aspartate [15]. Additionally, Clark et al. proposed that NAA could, under certain circumstances, serve as a reservoir for Glu [20]. A growing body of evidence continues to underline the involvement of NAA in BD pathophysiology [21,22,23,24].

Therefore, in this study, we conducted a meta-analysis to determine whether BD patients have alterations in NAA levels in various regions of the prefrontal cortex (dorsolateral prefrontal cortex (dlPFC), ventrolateral prefrontal cortex (vlPFC), medial prefrontal cortex (mPFC), white matter prefrontal cortex (wmPFC)), ACC, or hippocampi compared to healthy controls. In addition to NAA, we compared levels of Glu, glutamine (Gln), and Glx (essentially corresponding to the sum of Glu and Gln levels) between BD patients and healthy subjects within the various regions of the brain mentioned above.

## 2. Materials and Methods

### 2.1. Protocol Registration

This study was carried out following the Preferred Reporting Items for Systematic Reviews and Meta-Analyses (PRISMA) statement.

The full protocol was uploaded to the International Prospective Register of Systematic Reviews (CRD42020182638). 

### 2.2. Study Search

The search was performed using Medline, Embase, and PsycInfo. All studies published before 18 November 2021 were included.

The following search equation was used in the All Fields mode: Bipolar AND (MRS OR « Magnetic Resonance Spectroscopy » OR « Magnetic Resonance Spectroscopies »). The search was supplemented by bibliographic and textbook cross-referencing, as well as reviewing previous meta-analyses and systematic reviews, in order to avoid missing any potential studies for inclusion.

### 2.3. Study Selection

#### 2.3.1. Publication Type

The selected studies were required to be written in English and provide complete articles in the form of cross-sectional studies or randomized controlled trials. Additionally, the selected studies were required to include both a group of BD patients and a group of healthy controls (HCs).

#### 2.3.2. Inclusion and Exclusion Criteria

Studies were included if

(1) Patients met the Diagnostic and Statistical Manual of Mental Disorders (DSM) 3rd, 4th, or 5th edition criteria for BD or the International Classification of Disease diagnostic (ICD) criteria for BD.
(2) HCs did not have any mental illnesses according to these same references.
(3) BD patients met the criteria for a major depressive episode or clinical remission.
(4) BD patients and HCs were between the ages of 18 and 65 years.
(5) The regions of interest (ROI) targeted were the mPFC, dlPFC, vlPFC or wmPFC, ACC, and/or hippocampi.

Studies were excluded if

(1) BD patients and HCs had any other history of psychiatric or neurological conditions, head injuries, or addictive co-morbidities (except for smoking).
(2) The ^1^H-MRS technique was not used.
(3) The following metabolites were not quantified: NAA, Glu, Glx, and Gln.
(4) None of the ROIs were targeted.

### 2.4. Data Extraction

Two authors (JC and EA) jointly determined the keywords and screened the abstracts and titles according to the inclusion and exclusion criteria. The authors then evaluated the full texts independently in order to determine eligible studies. Any study exclusions were justified in accordance with the PRISMA criteria. Disagreements between authors on whether or not to include a study were resolved through discussion. Data were extracted by JC and EA independently, stored in an Excel spreadsheet, and compared. Again, disagreements were resolved through discussion. If different publications reported data from the same population, we included data from the publication with the larger sample size. If data or necessary information were missing from a published article, the authors of the studies were contacted for retrieval.

### 2.5. Quality Assessment

The quality of the original studies was assessed using the Newcastle–Ottawa Quality Assessment Scale after arrangement for a cross-sectional study design, similar to the meta-analysis conducted by Moriguchi et al. [25].

### 2.6. Statistical Analyses

Statistical analyses were performed with the Stata software (version 15, StataCorp, College Station, TX, USA). The meta-analysis considered between- and within-study variability. To address the non-independence of data due to study effects, random-effects models [26] were preferred over the usual statistical tests to assess standardized mean differences (SMDs) and their 95% confidence intervals. Means and standard deviations were compiled when available or estimated using Hozo et al. when median and interquartile ranges were reported [27]. SMDs were interpreted according to Cohen, where <0.2 was considered trivial, 0.2–0.3 was considered small, 0.5–0.8 was considered moderate, and >0.8 was considered large [28,29].

At the lateral ROIs (wmPFC, dlPFC, vlPFC, or hippocampi), the calculation of SMDs was performed for both the right and left hemispheres. At the level of medial ROIs (ACC or mPFC) many, although not all, studies used a single voxel spanning both hemispheres, thereby precluding separate results for each hemisphere. Thus, similar to Moriguchi et al., data for the left lobe were used at the level of medial ROIs when data from bilateral lobes were reported separately, as the left lobe was examined in most studies [25].

The same statistical approach was adapted for stratified analyses. Heterogeneity in the study results was assessed using forest plots and the I^2^ statistic, which is typically considered low at 25%, modest at 25% to 50%, and high when above 50% [30]. Publication bias was assessed by funnel plots and confidence intervals for each assessment method, one at a time, due to their great effects on heterogeneity. 

Subgroup analyses were then performed. First, we divided the ACC studies into perigenual ACC (composed of pre- and subgenual regions) and dorsal ACC (sometimes also called the MCC). Second, we performed comparisons for each region according to the method used to quantify the metabolites (absolute vs. relative), since creatine relative quantification is a less accurate technique.

To check the robustness of the results, sensitivity analyses were performed, excluding studies that would not be evenly distributed around the base of the funnel. More precisely, for our significant results that contained more than two studies, we performed a leave-one-out meta-analysis. As studies commonly produce exaggerated effect sizes, which may distort the overall results, leave-one-out meta-analysis is a useful statistical approach to (i) investigate the influence of each study on the overall effect-size estimate and (ii) identify influential studies.

## 3. Results

### 3.1. Characteristics of Included Studies

**Figure 1:**
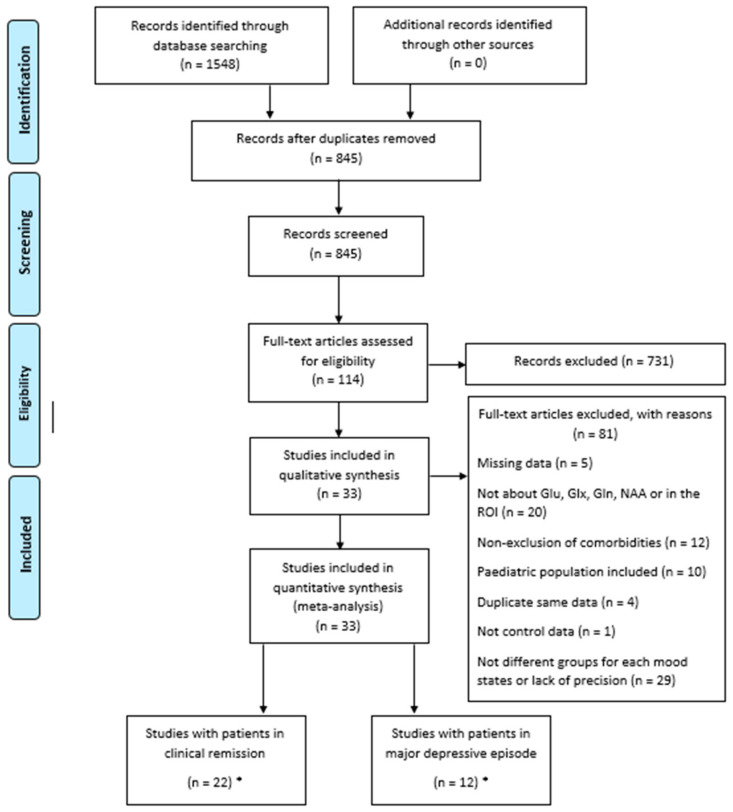
Preferred reporting items for systematic reviews and meta-analyses (PRISMA) diagram for study search. * One study featured two groups (clinical remission and depressive episode) and thus was counted twice, once in each category.

The search identified 33 studies [31,32,33,34,35,36,37,38,39,40,41,42,43,44,45,46,47,48,49,50,51,52,53,54,55,56,57,58,59,60,61,62,63] (Figure 1), which included a total of 800 HCs and 873 BD patients. Among these 33 studies, 11 studies included patients in a major depressive episode (238 patients), 21 studies included BD patients in clinical remission (603 patients), and one study combined BD patients in a major depressive episode and in clinical remission (22 patients in each group). The characteristics of these studies are described in Table 1 and Table 2.

In depressed patients, ten studies examined NAA (83%), including four examining the wmPFC, two examining the dlPFC, one examining the mPFC, eight examining the ACC, and three examining the hippocampi. Four studies examined Glu (33%), including three examining Glu in the ACC and one examining Glu in the hippocampi. Five studies examined Glx levels (42%), with two examining the dlPFC, one examining the mPFC, and four examining the ACC.

In remitted patients, nineteen studies examined NAA (86%), including four examining the wmPFC, five examining the dlPFC, one examining the mPFC, one examining unspecified the PFC, seven examining the ACC, and eight examining the hippocampi. Seven studies examined Glu (32%), including four examining Glu in the wmPFC, two examining Glu in the dlPFC, one examining Glu in the mPFC, one examining Glu in unspecified PFC, seven examining Glu in ACC, and two examining Glu in the hippocampi. Two studies examined Glx (9%), with four examining Glx in the wmPFC, two examining Glx in the dlPFC, one examining Glx in the mPFC, one examining Glx in unspecified PFC, seven examining Glx in ACC, and three examining Glx in hippocampi. Additionally, three studies examined Gln (14%), all in the ACC.

Four studies used a magnetic field of 1.5 Tesla on depressed patients (33% of studies with depressed patients) and nine studies used a magnetic field of 1.5 Tesla on remitted patients (41% of studies with remitted patients). A magnetic field of 3 Tesla was used in seven studies on depressed patients (58%) and nine studies on remitted patients (41%). Only one study used a 4 Tesla magnetic field on depressed patients (8%), and two used a 4 Tesla magnetic field on remitted patients (9%). Two studies involving patients in remission did not give the intensity of the magnetic fields used (see Table 1).

Only cross-sectional studies were found, and there was no RCT.

The Newcastle–Ottawa Scale score ranged from 3 to 6, with the average being 4.48, suggesting that the quality of the included studies was good on average (see Supplementary Tables S1 and S2).

### 3.2. Meta-Analysis

#### 3.2.1. BD Patients in Major Depressive Episode

**Figure 2:**
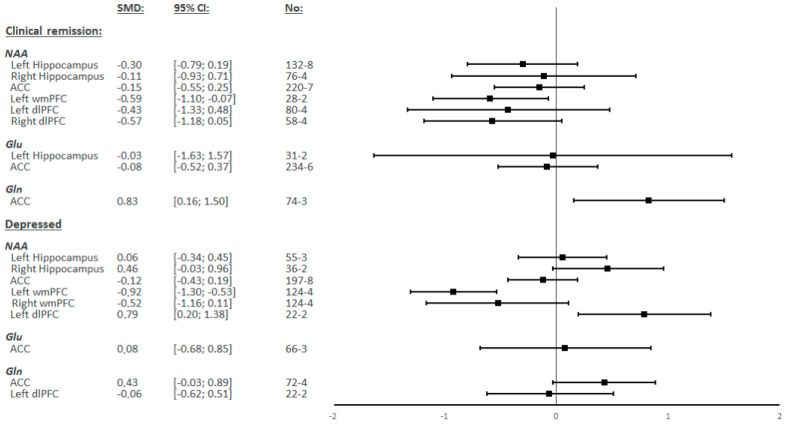
Summary of Standardized Mean Differences (SMDs) and their confidence intervals (95% CI) for each metabolite in each of the different regions by the mood status of bipolar patients.

**Figure 3:**
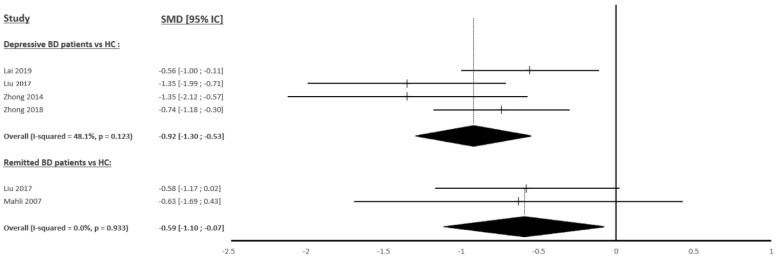
Studies Standardized Mean Differences (SMDs) of N-acetylaspartate differences between bipolar patients and controls in the left white matter prefrontal cortex [44,46,47,61,62].

NAA levels in the wmPFC were measured in four studies, including 124 BD patients and 118 HCs. In the left hemisphere, there were significantly lower levels of NAA in BD patients compared to the controls (SMD = −0.92; 95% CI: −1.30 to −0.53; I^2^ = 48.1%; *p* = 0.123) (Figure 2 and Figure 3). In the right hemisphere, there were no significant differences observed (SMD = −0.52; 95% CI: −1.16 to 0.11; I^2^ = 82.1%; *p* = 0.001) (Figure 2 and Supplementary Figure S12). 

NAA levels in the dlPFC were measured in two studies, including 22 BD patients and 27 HCs in the left hemisphere, but no study measured these levels in the right hemisphere. In the left hemisphere, there were significantly higher levels of NAA observed in BD patients compared to the controls (SMD = 0.79; 95% CI: 0.20 to 1.38; I^2^ = 0.0%; *p* = 0.336) (Figure 2 and Supplementary Figure S13). 

NAA levels in the other regions, as well as Glu, Glx, and Gln levels in all analyzed regions, showed no significant differences between BD patients and HCs (Figure 2 and Supplementary Figure S3, Figure S4, Figure S7, Figure S9A, Figure S10A and Figure S16).

#### 3.2.2. BD Patients in Clinical Remission

NAA levels in the wmPFC were measured in two studies including 31 BD patients and 33 HCs in the left hemisphere and in one study in the right hemisphere. In the left hemisphere, there were significantly lower levels of NAA observed in BD patients compared to the controls (SMD = −0.59; 95% CI: −1.10 to −0.07; I^2^ = 0.0%; *p* = 0.933) (Figure 2 and Figure 3). The study by Liu et al. [46] was the only research to measure NAA in the right wmPFC but did not find any significant difference.

Gln levels in ACC were measured in three studies including 85 BD patients and 103 HCs. There were significantly higher levels of Gln observed in BD patients compared to the controls (SMD = 0.83; 95% CI: 0.16 to 1.50; I^2^ = 66.6%; *p* = 0.050) (Figure 2 and Supplementary Figure S6A).

NAA levels in the other regions, as well as Glu and Glx levels in all regions, showed no significant differences between BD patients and HCs (Figure 2 and Supplementary Figure S1, Figure S2A, Figure S5A, Figure S8A, Figure S11A, Figure S14 and Figure S15A).

### 3.3. Sensitivity-Analysis and Subgroup Analyses

We performed a leave-one-out sensitivity analysis on our significant results and found that our results were robust. 

Our results obtained in the ACC were further analyzed by dividing our studies into two groups according to mood state: those whose ROI was the perigenual part of the ACC, and those whose ROI was the dorsal part of the ACC. Only the study by Croarkin et al. [36] could not be classified, as it did not provide the exact positioning of the ROI, and no clarification was obtained from the authors. No significant difference was found in either subgroup for any metabolite between BD patients (remitted or depressed) and HCs (Supplementary Figures S8C, S10C,D and S11C,D).

When possible, other subgroup analyses were also conducted for each metabolite in each region based on varying reference methods (absolute quantification vs. relative quantification). No significant differences were found in these analyses (Supplementary Figures S2B, S5B, S6B, S8B, S9B, S10B, S11B and S15B).

## 4. Discussion

Our meta-analysis aimed to determine whether the quantification of NAA, Glu, Gln, and/or Glx levels in the brains of BD patients without comorbidities could be used to better understand the neurobiological mechanisms of BD. Our results show that NAA levels in BD patients were significantly decreased in the left wmPFC during depressive and euthymic periods, as well as significantly increased in the left dlPFC during depressive periods. Meanwhile, Gln levels were significantly increased in the ACC in BD patients during euthymic periods when compared to HCs. The levels of NAA, Gln, Glu, and Glx were not statistically different between BD patients and HCs in any other regions analyzed.

The decrease in NAA levels in the wmPFC observed in BD patients was one of the most interesting findings of our study, even though the quantification was relative with creatine and not absolute. NAA is produced in the neural mitochondria from L-aspartate and acetyl coenzyme A. Studies have shown that NAA production is closely correlated with that of ATP and mitochondrial oxygen consumption [64,65,66], suggesting that NAA levels may reflect the integrity of mitochondrial energy metabolism [15,16]. Through energy production, as well as many other mechanisms, mitochondria are involved in neuroplasticity processes, development, and axonal regeneration [67,68]. The decrease in NAA localized within the white matter of the prefrontal cortex is, therefore, consistent with the alterations in neuroplasticity and synaptic plasticity found in BD patients [69]. NAA plays a unique role in the lipid synthesis of myelin sheaths since NAA allows the transfer of acetate groups from the neurons to the oligodendrocytes [15,16]. Thus, in addition to the abnormalities of energy production, neuroplasticity and synaptic plasticity may be indirectly impaired within the wmPFC via decreased NAA levels in BD patients. This decrease could also generate direct impairments of synaptic transmission and explain the T2 and FLAIR hypersignals found by MRI [8], especially since the voxels of the studies included in our meta-analysis appeared to be located in the same region as the hypersignals (deep white matter of the PFC). Unfortunately, due to the location of the voxels where numerous nerve fibers with different destinations pass, it is difficult to link our outcomes with functional abnormalities known in BD. A decrease in NAA was observed during both depressive periods and clinical remission, but our results in the left hemisphere for patients in clinical remission were clearly influenced by one of the two studies. Therefore, it will be interesting for future studies to confirm this tendency and evaluate whether NAA levels vary according to the duration of clinical remission. Furthermore, it remains necessary to test whether decreased NAA levels are also present during manic episodes. 

NAA levels were also significantly increased in the left dlPFC of depressed BD patients compared to HCs. Although this increase was also found by Kraguljac et al. in their previous meta-analysis [17], the functional and anatomical MRI data did not specifically find abnormalities in this region during depressive episodes [70,71]. One explanatory hypothesis could be related to the effects of lithium or valproate medication on the included BD patients [49]. Indeed, these treatments lead to an increase in Bcl-2 protein in the frontal cortex, which is a protein located in the mitochondrial membrane and involved in mitochondrial oxidation–reduction processes, as well as in neuroprotection [72,73]. However, ^1^H-MRS could also reveal anomalies not found using other MRI techniques. If these results are not explained by the effects of medications, it will be relevant to more precisely investigate this region based on the hypothesis that it may over-function in bipolar depression.

Regarding Glu and Gln metabolites, very few studies met our inclusion criteria in the PFC, resulting in a meta-analysis that could include only Glx in the dlPFC and did not find any significant differences between depressed BD patients and HCs. It would be interesting to be able to include more studies feature additional regions of the PFC.

In the two hippocampi, there were no significant differences found between BD patients and HCs for any of the investigated metabolites. The hippocampal formation is a very plastic and vulnerable brain region in which anomalies in the sizes of neurons and a reduction in the number of glial cells was identified in BD patients [74,75]. Therefore, we expected to discover decreased NAA levels in BD patients, as post-mortem studies have found mitochondrial dysfunction in the hippocampi of these patients, including decreased expression of nuclear mRNA encoding mitochondrial proteins [76]. Alterations of proteins involved in glycogenogenesis, glycogenolysis, and mitochondrial energy functions have also been identified [77]. Our findings differ from those of Kraguljac et al., who found a significant decrease in NAA/Cr in the hippocampi [17], even though, as explained previously, this meta-analysis mixed patients of various mood states. Conversely, an increase in Glu levels was also expected, since stress and glucocorticoids, which are very present in BD patients, can increase the concentrations of extracellular Glu in the hippocampi [78] alongside high levels of expression of the GCP II enzyme in the hippocampi of BD patients, which hydrolyzes NAAG into Glu in the glial cells [24]. However, ^1^H-MRS techniques do not measure glutamatergic transmission, which is a small part of cerebral Glu stocks, but instead measure total brain Glu, which is used in many brain functions other than neurotransmission [19]. This factor could explain the absence of significant increases in glutamatergic metabolites in the hippocampi of BD patients. Another explanation could be the difficulty of obtaining quality spectra in this region due to its proximity to air–tissue interfaces.

In the ACC, no significant differences were found between BD patients and HCs, regardless of the metabolites studied. Scotti-Muzzi et al. conducted a meta-analysis comparing several neurometabolites between BD patients and HCs, specifically targeting the ACC region. Although our meta-analysis included several studies that were not included in the analyses of Scotti-Muzzi et al. due to their more recent publication dates, our results for NAA, Glu, and Gln levels remain consistent with the results of this previous study [79]. Regarding Glx levels, the results from our study were only available for BD patients in depressive episodes, and these results differ from those of Scotti-Muzzi et al. [79]. However, it is important to specify that the outcomes of Scotti-Muzzi et al. could not be evaluated according to the mood state, as the number of studies available was lower than three. This increase in Glx levels could, therefore, be due to the non-depressive periods of the disease and may be caused, in particular, by the significant increase in Gln levels found in BD patients in the euthymic period. In another meta-analysis, Taylor et al. compared Glx levels in the ACC of BD patients in major depressive episodes to HCs [80]. Although the authors included studies that did not fully meet our inclusion criteria (including a study with adolescent BD patients, another with some patients in a mixed episode, and one study in which the ROI encompassed part of the mPFC), they also did not find any significant differences. The ACC is a complex region encompassing various sub-regions with specific connectivity and functions, the limits of which are mainly derived from the Brodmann classification [81,82]. Brain imaging studies usually divide the ACC into two main parts: the peri-genual ACC and the dorsal ACC (also commonly called the midcingulate cortex (MCC)). In our study, we performed a subgroup analysis to see if there were variations in the results according to the parts of the ACC being examined. However, we did not find any significant differences. 

This meta-analysis has some limitations. First, although the meta-analysis analyzed data region by region, as well as by sub-region in the ACC, voxel sizes and ROIs were not similar between the included studies. These differences may have skewed the results of the ROI analyses. Second, the magnetic field strength, ^1^H-MRS editing techniques, and echo-time also differed between included studies. Third the Cramer–Rao lower bound (CRLB), which is a marker of spectrum quality, was rarely precise in the different studies. Fourth, because many studies did not detail the patients’ treatments, we could not perform meta-regression by treatment to assess the influences of different approaches. Some studies showed that treatments can partially modify the concentrations of certain metabolites in certain regions [24]. However, notably, for our primary outcome of decreased NAA in the left wmPFC in depressed patients, BD patients were not medicated in three of the four included studies, yet the heterogeneity was described as modest. Fifth, because of the lack of data and inconsistency in the scales used, we could not verify whether metabolite concentrations were dependent on the intensity of depression. Likewise, we could not verify whether metabolite concentrations were dependent on the age of onset or number of mood episodes. These factors potentially influenced our results.

In summary, in the present study, we observed decreased NAA levels in the wmPFC of both euthymic and depressed BD patients. These results emphasize the role of mitochondrial energy metabolism in neuroplasticity and synaptic plasticity in BD.

## Figures and Tables

**Table 1:** Characteristics of studies including BD patients in clinical remission.

Study (Year)	Field Strength (Tesla)	TE (ms)	TR (ms)	Acquisition Sequence	CRLB Threshold	Creatine Scaling	Patients (n)	Controls (n)	Age	Gender (Male/Female)	Metabolite	ROI
Amaral (2006) [31]	1.5	144	1500	PRESS		Cr-scaling	13	15	34.54	6/7	NAA	pACC
Brady (2012) [33]	4	30–500	2000	JPRESS	<25%	Cr-scaling	7	6	39.70	3/4	NAA, Glu	pACC
Colla (2009) [34]	3	80	3000	PRESS			21	19	54.20	10/11	NAA, Glu	Hipp
Corcoran (2020) [35]	3	80	3000	PRESS	<15%		17	41	44.64	11/6	Glu	dACC, dlPFC
Cumurcu (2008) [37]	X	136	2000	PRESS		Cr-scaling	10	10	33.10	5/5	NAA	Hipp, dlPFC
Deicken (2003) [38]	1.5	135	1800				15	20	39.30	15/0	NAA	Hipp
Ehrlich (2015) [39]	3	80	3000	PRESS	<20%		21	42	45.90	8/13	NAA, Glu, Gln	Hipp, dACC
Haarman (2016) [40]	3	144	2000	PRESS	<20%		22	24	44.50	10/12	NAA	Hipp
Iosifescu (2009) [41]	4	30	2000	PRESS	<15%		20	10	40.70	14/6	NAA	Hipp
Kalayci (2012) [42]	1.5	35 and 144	3000	PRESS			15	15	38.87	9/6	NAA	dlPFC
Kubo (2016) [43]	3	18	5000	STEAM			14	23	45.00		NAA, Glu, Gln	dACC
Liu (2017) [46]	3	144	1000	PRESS		Cr-scaling	22	24	26.82	5/17	NAA	wmPFC
Mahli (2007) [47]	1.5	80	1500	STEAM			9	9	40.78	2/7	NAA	pACC, wmPFC
Molina (2007) [50]	1.5	136	1500	PRESS		Cr-scaling	13	10	37.80	13/0	NAA	dlPFC
Rocha (2015) [51]	1.5	30	1500	PRESS			21	22	42.00	5/16	NAA	mOFC
Scherk (2008) [52]	1.5	30	1500	PRESS		Cr-scaling	13	13	31.45	6/7	NAA	Hipp
Scherk (2009) [53]	1.5	30	1500	PRESS		Cr-scaling	33	29	43.86	15/18	NAA	dACC, dlPFC
Senaratne (2009) [54]	3	35	2000	PRESS	<20%		12	12	42.10	3/9	NAA, Glx	Hipp, mPFC
Soiero-De Souza (2015) [56]	3	31–231	1600	JPRESS	<20%	Cr-scaling	50	38	31.70	19/31	Glu, Gln	dACC
Soeiro-De-Souza (2018) [57]	3	80	1500	PRESS	<20%	Cr-scaling	128	80	32.04	42/86	Glu, Glx	pACC
Soiero-De Souza (2018) [58]	3	80	1500	PRESS	<20%		129	79	32.00	44/85	NAA	pACC
Winberg (2000) [59]	1.5	35	2000	PRESS		Cr-scaling	20	20	37.90	9/11	NAA	dlPFC

BD = bipolar disorder; TE = time to echo; TR = repetition time; CRLB = Cramer–Rao lower bound; ROI = region of interest; NAA = N-acetylaspartate; Glu = glutamate; Gln = glutamine; mOFC = medial orbitofrontal cortex; dlPFC = dorsolateral prefrontal cortex; mPFC = medial prefrontal cortex; wmPFC = white matter PFC; pACC = perigenual anterior cingulate cortex; dACC = dorsal anterior cingulate cortex; Hipp = hippocampus.

**Table 2:** Characteristics of studies including BD patients in a major depressive episode.

Study (Year)	Field Strength (Tesla)	TE (ms)	TR (ms)	Acquisition Sequence	CRLB Threshold	Creatine Scaling	Patients (n)	Controls (n)	Age	Gender (Male/Female)	Metabolite	ROI
Atmaca (2012) [32]	1.5					Cr-scaling	16	16	28.10	12/4	NAA	Hipp
Croarkin (2015) [36]	1.5	30	2000	L-COSY		Cr-scaling	15	9			Glu, Glx, NAA	ACC
Lai (2019) [44]	3	144	1000	PRESS		Cr-scaling	40	40	24.88	17/23	NAA	dACC, wmPFC
Li (2016) [45]	3	30	1500	PRESS	<20%		13	20	31.00	6/7	NAA, Glx	dACC, mPFC
Liu (2017) [46]	3	144	1000	PRESS		Cr-scaling	22	22	24.36	6/16	NAA	wmPFC
Mellen (2019) [48]	4	30–500	2000	JPRESS		Cr-scaling	23	14	62.00	14/9	NAA, Glu	pACC
Michael (2009) [49]	1.5	20	2500	STEAM	<20%		6	6	51.60	1/5	NAA, Glx	dlPFC
Smaragdi (2019) [55]	3	35	1500	PRESS			16	21	37.00	9/7	NAA, Glx	dACC, dlPFC
Zanetti (2014) [60]	3	35	1500	PRESS	<20%		19	17	28.70	6/13	NAA, Glu	Hipp
Zhong (2018) [61]	3	144	1000	PRESS		Cr-scaling	42	43	26.62	17/25	NAA	dACC, wmPFC
Zhong (2014) [62]	1.5	144	1000	PRESS		Cr-scaling	20	13	30.55	9/11	NAA	Hipp, pACC, wmPFC
Soiero-De-Souza (2021) [63]	3	80	1500	PRESS	<20%	Cr-scaling	28	28	28.30	7/21	NAA, Glx, Glu	ACC

BD = bipolar disorder; TE = time to echo; TR = repetition time; CRLB = Cramer–Rao lower bound; ROI = region of interest; NAA = N-acetylaspartate; Glu = glutamate; Gln = glutamine; dlPFC = dorsolateral prefrontal cortex; mPFC = medial prefrontal cortex; wmPFC = white matter PFC; pACC = perigenual anterior cingulate cortex; dACC = dorsal anterior cingulate cortex; Hipp = hippocampus.

## Supplementary Materials

The following supporting information can be downloaded at: https://www.mdpi.com/article/10.3390/ijms23168974/s1. References [35,42,73,83,84,85,86,87,88,89,90,91,92,93,94,95,96,97,98,99,100,101,102,103,104,105,106,107,108,109,110,111,112,113,114,115,116,117,118,119,120,121,122,123,124,125,126,127,128,129,130,131,132,133,134,135,136,137,138,139,140,141,142,143,144,145,146,147,148,149,150,151,152,153,154,155,156,157,158,159,160,161,162] are cited in the supplementary materials.
