# Supplementary material for: Glutamatergic and N-Acetylaspartate Metabolites in Bipolar Disorder: A Systematic Review and Meta-Analysis of Proton Magnetic Resonance Spectroscopy Studies
Source: Int J Mol Sci. 2022 Aug 11;23(16):8974. doi: 10.3390/ijms23168974 (PMC9409038; doi:10.3390/ijms23168974)

SUPPLEMENTARY TABLES:

Supplementary Table S1: The Newcastle-Ottawa Scale (NOS) results:

	Selection:				Comparability:		
Study and year:	Case definition:	Representativeness:	Selection of control:	Definition of control:	Age:	Other factor:	Total:
Rocha (2015)	1	0	1	1	0	0	3
Winberg (2000)	1	1	1	1	0	1	5
Senaratne (2009)	1	1	1	1	1	1	6
Scherk (2009)	1	0	1	1	0	0	3
Molina (2007)	1	1	1	1	0	0	4
Kalayci (2012)	1	0	1	1	1	1	5
Cumurcu (2008)	1	0	1	1	0	0	3
Corcoran (2020)	1	1	1	1	0	0	4
Mahli (2007)	1	0	1	1	1	1	5
Ehrlich (2015)	1	0	1	1	1	1	5
Soiero-De Souza (2015)	1	0	1	1	0	0	3
Soiero-De Souza (2018 [1])	1	1	1	1	0	0	4
Soeiro-De-Souza 20(18 [2])	1	1	1	1	0	0	4
Amaral (2006)	1	0	1	1	0	1	4
Brady (2012)	1	0	1	1	0	1	4
Kubo (2016)	1	1	1	1	1	1	6
Iosifescu (2009)	1	0	1	1	1	1	5
Colla (2009)	1	1	1	1	1	1	6
Haarman (2016)	1	1	1	1	1	1	6
Scherk (2008)	1	0	1	1	1	1	5
Deicken (2003)	1	0	1	1	0	1	4
Liu (2017)	1	0	1	1	1	1	5
Michael (2009)	1	0	1	1	1	1	5
Li (2016)	1	0	1	1	1	1	5

Smaragdi (2019)	1	1	1	1	0	0	4
Zhong (2014)	1	0	1	1	1	1	5
Zhong (2018)	1	0	1	1	1	1	5
Lai (2019)	1	0	1	1	1	1	5
Croarkin (2015)	1	1	1	1	0	0	4
Mellen (2019)	1	1	1	1	0	0	4
Zanetti (2014)	1	0	1	1	0	0	3
Atmaca (2012)	1	0	1	1	0	0	3
Soeiro-De-Souza (2021)	1	1	1	1	1	1	6

Supplementary Table S2: Excluded studies:

Name of the study	First autor	Reason for exlcusion
Lithium increases N-acetyl-aspartate in the human brain: in vivo evidence in support of bcl-2's neurotrophic effects? [73]	Moore	Missing data
Chronic treatment with both lithium and sodium valproate may normalize phosphoinositol cycle activity in bipolar patients. [83]	Silverstone	Not about Glu, Glx, Gln, NAA (on myo-I)
Brain choline concentrations may not be altered in euthymic bipolar disorder patients chronically treated with either lithium or sodium valproate. [84]	Wu	Not about Glu, Glx, Gln, NAA (on Cho)
Brain metabolic alterations in medication-free patients with bipolar disorder. [85]	Dager	Not different groups for each mood states or lack of precision
Lithium and valproic acid treatment effects on brain chemistry in bipolar disorder. [86]	Friedman	Not different groups for each mood states or lack of precision
1H magnetic resonance spectroscopy investigation of the dorsolateral prefrontal cortex in bipolar disorder patients. [87]	Brambilla	Not different groups for each mood states or lack of precision
Metabolic alterations in medication-free patients with bipolar disorder: a 3T CSF-corrected magnetic resonance spectroscopic imaging study. [88]	Port	Not different groups for each mood states or lack of precision
Brain GABA levels in patients with bipolar disorder. [89]	Kaufman	Not different groups for each mood states or lack of precision
Bipolar disorder comorbid with alcoholism: a 1H magnetic resonance spectroscopy study. [90]	Nery	Not different groups for each mood states or lack of precision
T2 relaxation time abnormalities in bipolar disorder and schizophrenia. [91]	Ongur	Not different groups for each mood states or lack of precision
Brain-derived neurotrophic factor val66met polymorphism affects prefrontal energy metabolism in bipolar disorder. [92]	Frey	Not different groups for each mood states or lack of precision
1) Abnormal cellular energy and phospholipid metabolism in the left dorsolateral prefrontal cortex of medication-free individuals with bipolar disorder: an in vivo 1H MRS study. [93]<br>2) Corrected values of brain metabolites for the article: 'Abnormal cellular energy and phospholipid metabolism in the left dorsolateral prefrontal cortex of medication-free individuals with bipolar disorder: an in vivo 1H MRS study'. [94]	Frey	Not different groups for each mood states or lack of precision
In vivo glutathione levels in young persons with bipolar disorder: a magnetic resonance spectroscopy study. [95]	Lagopoulos	Not about Glu, Glx, Gln, NAA (on GSH)
Investigating the role of glutathione in mismatch negativity: An insight into NMDA receptor disturbances in bipolar disorder. [96]	Chitty	Not about Glu, Glx, Gln, NAA (on GSH)
Perisylvian GABA levels in schizophrenia and bipolar disorder. [97]	Atagun	Not about Glu, Glx, Gln, NAA (on GABA)
Increased Brain Lactate During Depressive Episodes and Reversal Effects by Lithium Monotherapy in Drug-Naive Bipolar Disorder: A 3-T 1H-MRS Study. [98]	Machado-Vieira	Not about Glu, Glx, Gln, NAA (on Lactate)
Neurochemical metabolites in the medial prefrontal cortex in bipolar disorder: A proton magnetic resonance spectroscopy study. [99]	Ozdel	Non-exclusion of comorbidities
Hippocampal neurochemical markers in bipolar disorder patients following the first-manic episode: A prospective 12-month proton magnetic resonance spectroscopy study. [100]	Silveira	Pediatric population included

Evidence of altered membrane phospholipid metabolism in the anterior cingulate cortex and striatum of patients with bipolar disorder I: A multi-voxel (1)H MRS study. [101]	Cao	Not different groups for each mood states or lack of precision
Trait-related alterations of N-acetylaspartate in euthymic bipolar patients: A longitudinal proton magnetic resonance spectroscopy study. [102]	Aydin	Non-exclusion of comorbidities
Neurochemical alterations in anterior cingulate cortex in bipolar disorder: a proton magnetic resonance spectroscopy study (1H-MRS). [103]	Galinska	Not different groups for each mood states or lack of precision
Elevated Choline-Containing Compound Levels in Rapid Cycling Bipolar Disorder. [104]	Cao	Not different groups for each mood states or lack of precision
Diagnosis and body mass index effects on hippocampal volumes and neurochemistry in bipolar disorder. [105]	Bond	Pediatric population included
Unique prefrontal GABA and glutamate disturbances in co-occurring bipolar disorder and alcohol dependence. [106]	Prisciandaro	Not different groups for each mood states or lack of precision
In Vivo Brain Glycine and Glutamate Concentrations in Patients With First-Episode Psychosis Measured by Echo Time-Averaged Proton Magnetic Resonance Spectroscopy at 4T. [107]	Kim	Non-exclusion of comorbidities
Choline Compounds of the Frontal Lobe and Temporal Glutamatergic System in Bipolar and Schizophrenia Proton Magnetic Resonance Spectroscopy Study. [108]	Galinska	Not different groups for each mood states or lack of precision
In vivo imaging of oxidative stress and fronto-limbic white matter integrity in young adults with mood disorders. [109]	Hermens	Not about Glu, Glx, Gln, NAA (on GSH)
Type 2 diabetes mellitus: a potentially modifiable risk factor for neurochemical brain changes in bipolar disorders. [110]	Hajek	Not different groups for each mood states or lack of precision
Inflammation, Glutamate, and Cognition in Bipolar Disorder Type II: A Proof of Concept Study. [111]	King	Not different groups for each mood states or lack of precision
Proton magnetic resonance spectroscopic imaging of gray and white matter in bipolar-I and schizophrenia. [112]	Bustillo	Not different groups for each mood states or lack of precision
Proton magnetic resonance spectroscopy of the brain in schizophrenic and affective patients. [113]	Sharma	Not different groups for each mood states or lack of precision
The human brain resonance of choline-containing compounds is similar in patients receiving lithium treatment and controls: an in vivo proton magnetic resonance spectroscopy study. [114]	Stoll	Non-exclusion of comorbidities
Choline, myo-inositol and mood in bipolar disorder: a proton magnetic resonance spectroscopic imaging study of the anterior cingulate cortex. [115]	Moore	Not different groups for each mood states or lack of precision
Neuronal pathology in the hippocampal area of patients with bipolar disorder: a study with proton magnetic resonance spectroscopic imaging. [116]	Bertolino	Not different groups for each mood states or lack of precision
Chronic treatment with lithium, but not sodium valproate, increases cortical N-acetyl-aspartate concentrations in euthymic bipolar patients. [117]	Silverstone	Non-exclusion of comorbidities
Lithium and valproate protect against dextro-amphetamine induced brain choline concentration changes in bipolar disorder patients. [118]	Silverstone	Non-exclusion of comorbidities

Hippocampal neurochemical pathology in patients at first episode of affective psychosis: a proton magnetic resonance spectroscopic imaging study. [119]	Blasi	Pediatric population included
Magnetic resonance spectroscopic measurement of cerebral gamma-aminobutyric acid concentrations in patients with bipolar disorders. [120]	Wang	Not different groups for each mood states or lack of precision
Dopamine transporter genotype influences N-acetyl-aspartate in the left putamen. [121]	Scherk	Non-exclusion of comorbidities
Reduction in occipital cortex gamma-aminobutyric acid concentrations in medication-free recovered unipolar depressed and bipolar subjects. [122]	Bhagwagar	Not in the regions of interest
Brain gamma-aminobutyric acid (GABA) abnormalities in bipolar disorder. [123]	Brady	Not about Glu, Glx, Gln, NAA (on GABA)
Cortical glutathione levels in young people with bipolar disorder: a pilot study using magnetic resonance spectroscopy. [124]	Godlewska	Non-exclusion of comorbidities
Cluster analysis reveals abnormal hippocampal neurometabolic profiles in young people with mood disorders. [125]	Hermens	Not different groups for each mood states
Individual prediction of symptomatic converters in youth offspring of bipolar parents using proton magnetic resonance spectroscopy. [126]	Zhang	Pediatric population included
Functional magnetic resonance spectroscopy in patients with schizophrenia and bipolar affective disorder: Glutamate dynamics in the anterior cingulate cortex during a working memory task. [127]	Jelen	Not different groups for each mood states or lack of precision
Hippocampal 1H MRS in patients with bipolar disorder taking valproate versus valproate plus quetiapine. [128]	Atmaca	Not different groups for each mood states or lack of precision
SNAP-25 genotype influences NAA/Cho in left hippocampus. [129]	Scherk	Non-exclusion of comorbidities
Association of the brain-derived neurotrophic factor val66met polymorphism with magnetic resonance spectroscopic markers in the human hippocampus: in vivo evidence for effects on the glutamate system. [130]	Gruber	Non-exclusion of comorbidities
Neurochemical alteration in the caudate: implications for the pathophysiology of bipolar disorder. [131]	Shahana	Pediatric population included
Increased thalamic phospholipid concentration evident in bipolar I disorder. [132]	Howells	Non-exclusion of comorbidities
Magnetic resonance spectroscopy imaging of lactate in patients with bipolar disorder. [133]	Chu	Not different groups for each mood states or lack of precision
Hippocampal glutamatergic/NMDA receptor functioning in bipolar disorder: A study combining mismatch negativity and proton magnetic resonance spectroscopy. [134]	Chitty	Not different groups for each mood states or lack of precision
Dorsal Anterior Cingulate Lactate and Glutathione Levels in Euthymic Bipolar I Disorder: 1H-MRS Study. [135]	Soeiro-De-Souza	Not about Glu, Glx, Gln, NAA (on GSH and Lactate)
ACC Glu/GABA ratio is decreased in euthymic bipolar disorder I patients: possible in vivo neurometabolite explanation for mood stabilization. [136]	Scotti-Muzzi	Duplicate same data
Neurochemical effects of quetiapine in patients with bipolar mania: a proton magnetic resonance spectroscopy study. [137]	Adler	Missing data
(1)H-MRS of hippocampus in patients after first manic episode. [138]	Gigante	Pediatric population included
Bcl-2 rs956572 polymorphism is associated with increased anterior cingulate cortical glutamate in euthymic bipolar I disorder. [139]	Soeiro-De-Souza	Duplicate same data

Investigation of Heschl's gyrus and planum temporale in patients with schizophrenia and bipolar disorder: a proton magnetic resonance spectroscopy study. [140]	Atagun	Not in the regions of interest
Neurochemical differences between bipolar disorder type I and II in superior temporal cortices: A proton magnetic resonance spectroscopy study. [141]	Atagun	Not in the regions of interest
Increased thalamic N-acetylaspartate in male patients with familial bipolar I disorder. [142]	Deicken	Not in the regions of interest
Genetic variant in SLC1A2 is associated with elevated anterior cingulate cortex glutamate and lifetime history of rapid cycling. [143]	Veldic	Not control data
Large positive effect of lithium on prefrontal cortex N-acetylaspartate in patients with bipolar disorder: 2-centre study. [144]	Hajek	Missing data
Increased anterior cingulate/medial prefrontal cortical glutamate and creatine in bipolar depression. [145]	Frye	Not in the regions of interest (PFC and ACC in an unique ROI)
Neurochemical abnormalities in unmedicated bipolar depression and mania: a 2D 1H MRS investigation. [146]	Xu	Missing data
Biochemical changes in the cingulum in patients with schizophrenia and chronic bipolar disorder. [147]	Sarramea	Not in the regions of interest (ACC and PFC in a unique ROI)
A Longitudinal (6-week) 3T (1)H-MRS Study on the Effects of Lithium Treatment on Anterior Cingulate Cortex Metabolites in Bipolar Depression. [148]	Machado-Vieira	Duplicate same data
Association of altered thyroid hormones and neurometabolism to cognitive dysfunction in unmedicated bipolar II depression [149]	Lai	Pediatric population included
Altered brain creatine cycle metabolites in bipolar I disorder with childhood abuse: A 1 H magnetic resonance spectroscopy study [50]	Soares Bio	Duplicate same data
Choline-containing compounds detected by proton magnetic resonance spectroscopy in the basal ganglia in bipolar disorder. [151]	Kato	Not in the regions of interest
Quantitative proton magnetic resonance spectroscopy of the basal ganglia in patients with affective disorders [152]	Hamakawa	Not in the regions of interest
Proton magnetic resonance spectroscopy of the lenticular nuclei in bipolar I affective disorder. [153]	Ohara	Not in the regions of interest
Biochemical abnormalities in basal ganglia and executive dysfunction in acute- and euthymic-episode patients with bipolar disorder: A proton magnetic resonance spectroscopy study. [154]	Lai	Not in the regions of interest
The characteristic of cognitive impairments in patients with bipolar II depression and its association with N-acetyl aspartate of the prefrontal white matter [155]	Zhong	Missing data
Proinflammatory Cytokines Predict Brain Metabolite Concentrations in the Anterior Cingulate Cortex of Patients With Bipolar Disorder [156]	Poletti	Not different groups for each mood states or lack of precision
Quantitative proton magnetic resonance spectroscopy of the bilateral frontal lobes in patients with bipolar disorder. [157]	Hamakawa	Not in the regions of interest (lack of precision of the ROI in the PFC)
Weight gain as a risk factor for progressive neurochemical abnormalities in first episode mania patients: a longitudinal magnetic resonance spectroscopy study. [158]	Bond	Pediatric population included

Increased Glutamate Plus Glutamine in the Right Middle Cingulate in Early Schizophrenia but Not in Bipolar Psychosis: A Whole Brain 1H-MRS Study. [159]	Bustillo	Pediatric population included
Evidence of altered metabolism of cellular membranes in bipolar disorder comorbid with post-traumatic stress disorder. [160]	Jabbari-Zadeh	Not different groups for each mood states or lack of precision
Salience network glutamate and brain connectivity in medication-naïve first episode patients – A multimodal magnetic resonance spectroscopy and resting state functional connectivity MRI study [161]	Maximo	Pediatrics population included
Mapping Disease Course Across the Mood Disorder Spectrum Through a Research Domain Criteria Framework [162]	Whitton	Not exclusion of comorbidities

Hippocampus:

Supplementary Figure S1: Studies SMDs of glutamate differences between euthymic BD patients and controls in the left hippocampus:

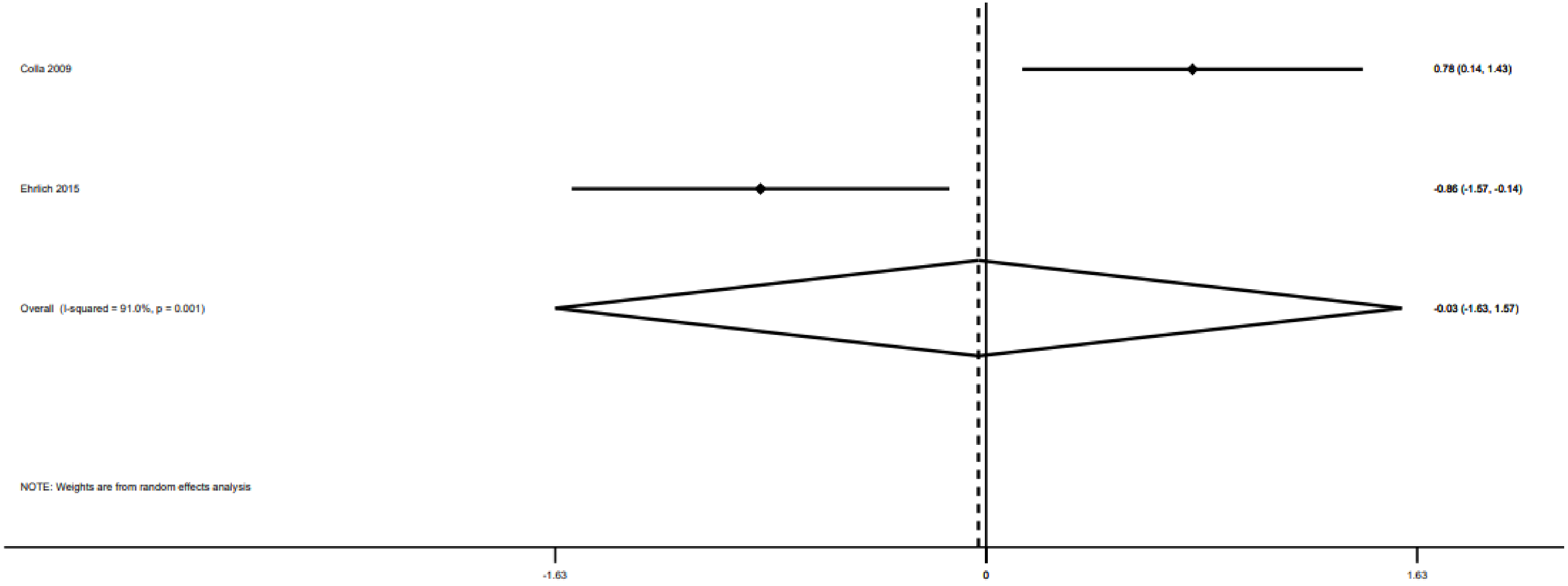

Supplementary Figure S2A: Studies SMDs of N-acetylaspartate differences between euthymic BD patients and controls in the left hippocampus:

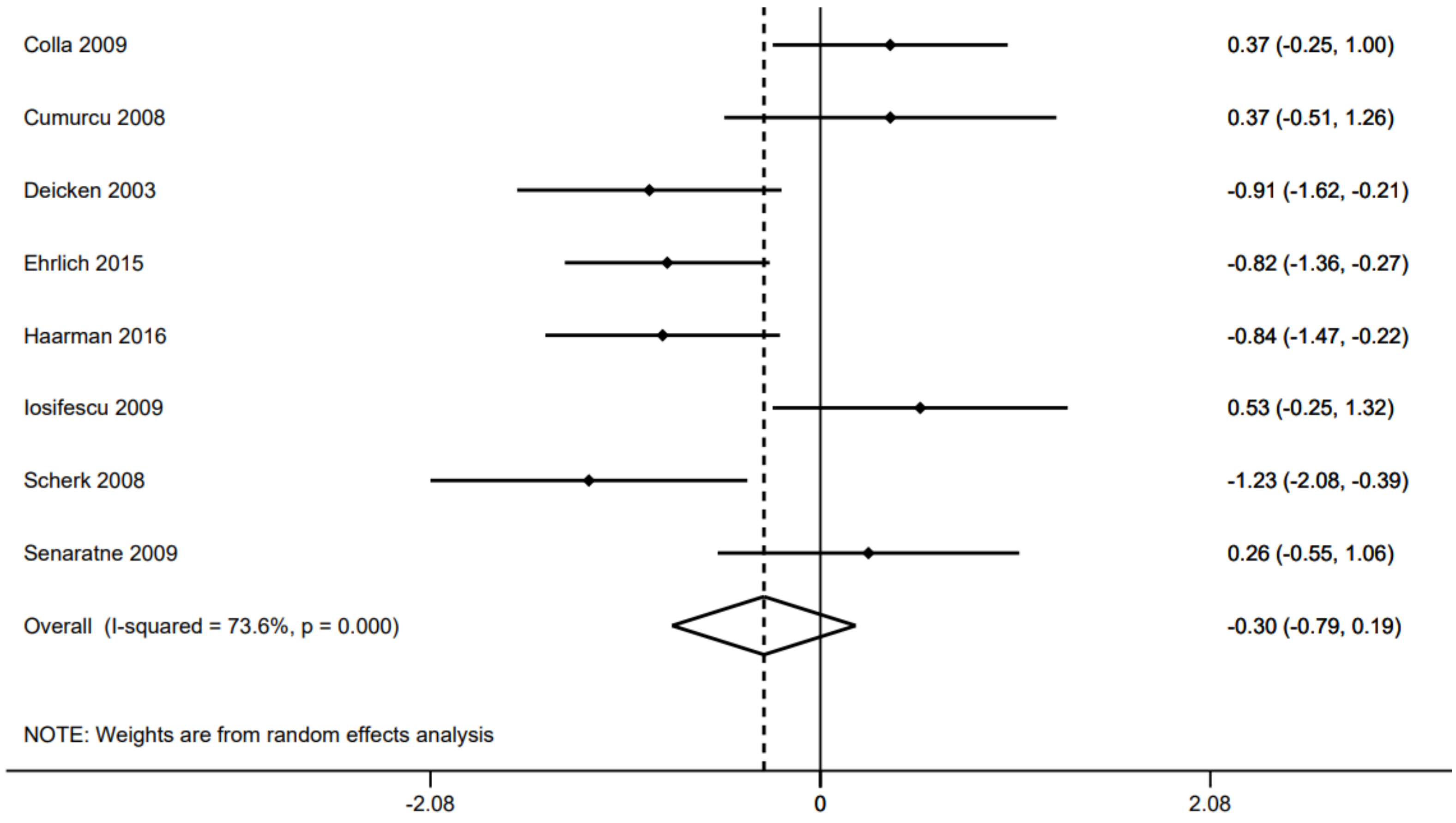

Supplementary Figure S2B: Studies SMDs of N-acetylaspartate differences between euthymic BD patients and controls in the left hippocampus according to quantification methodology:

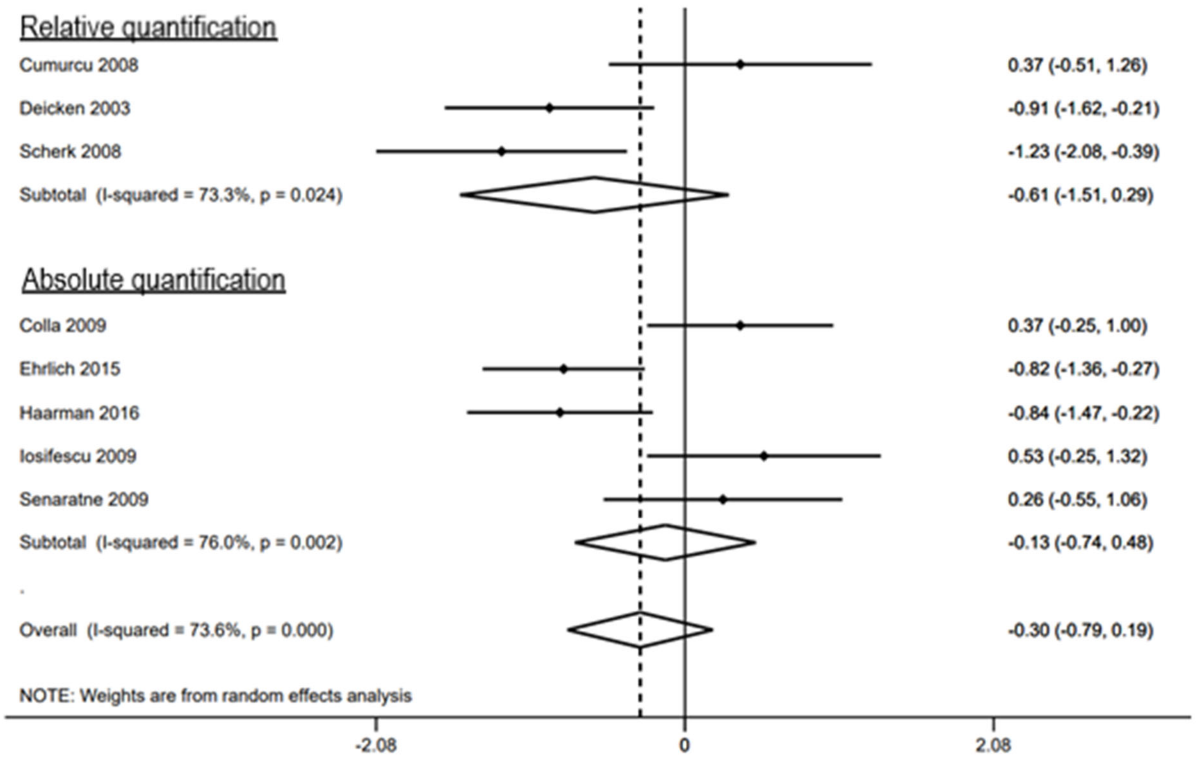

Supplementary Figure S3: Studies SMDs of N-acetylaspartate differences between depressed BD patients and controls in the left hippocampus:

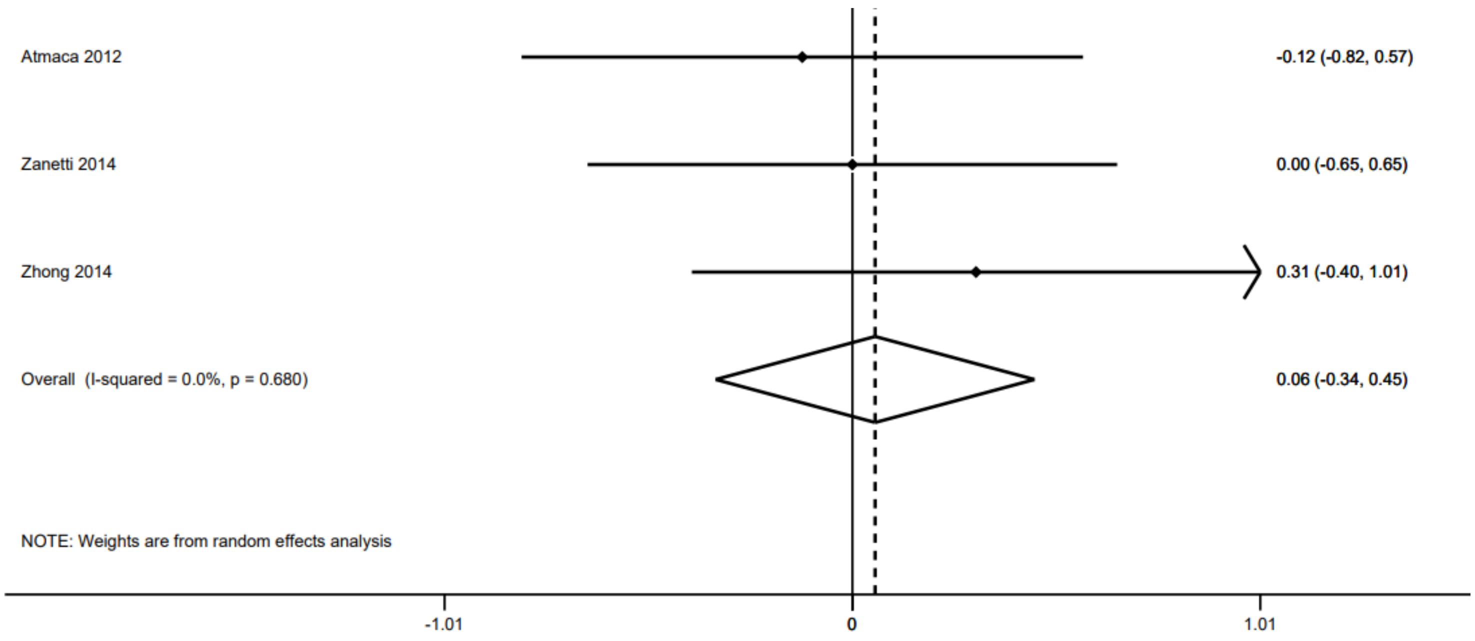

Supplementary Figure S4: Studies SMDs of N-acetylaspartate differences between depressed BD patients and controls in the right hippocampus:

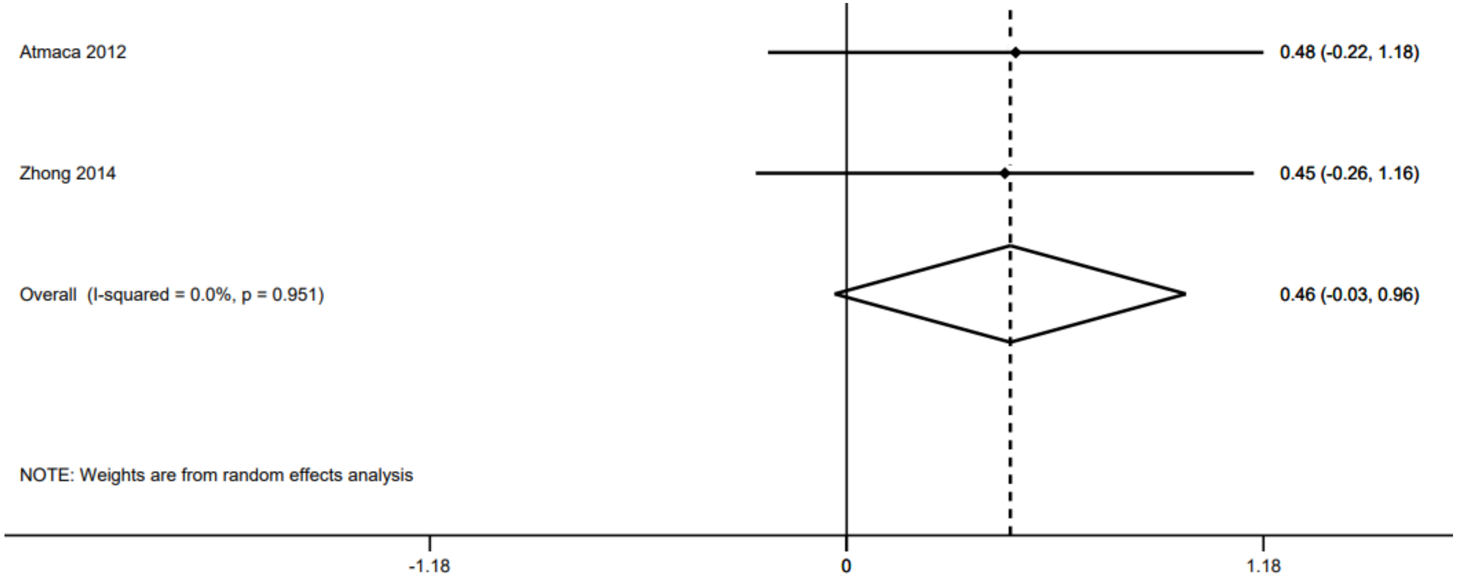

Atmaca 2012
0.48 (-0.22, 1.18)
Zhong 2014
0.45 (-0.26, 1.16)
Overall (I-squared = 0.0%, p = 0.951)
0.46 (-0.03, 0.96)
NOTE: Weights are from random effects analysis
-1.18
0
1.18

Supplementary Figure S5A: Studies SMDs of N-acetylaspartate differences between euthymic BD patients and controls in the right hippocampus:

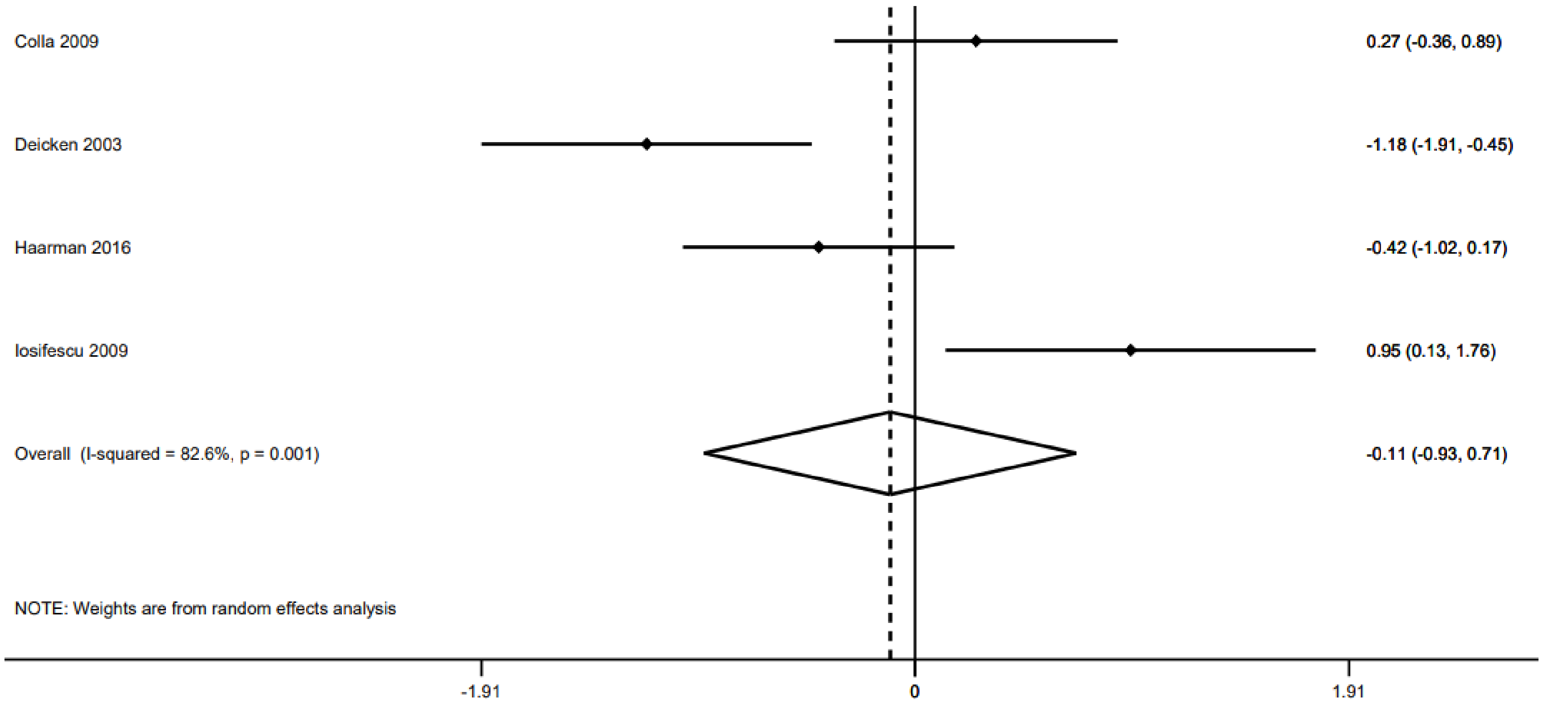

Supplementary Figure S5B: Studies SMDs of N-acetylaspartate differences between euthymic BD patients and controls in the right hippocampus according to quantification methodology:

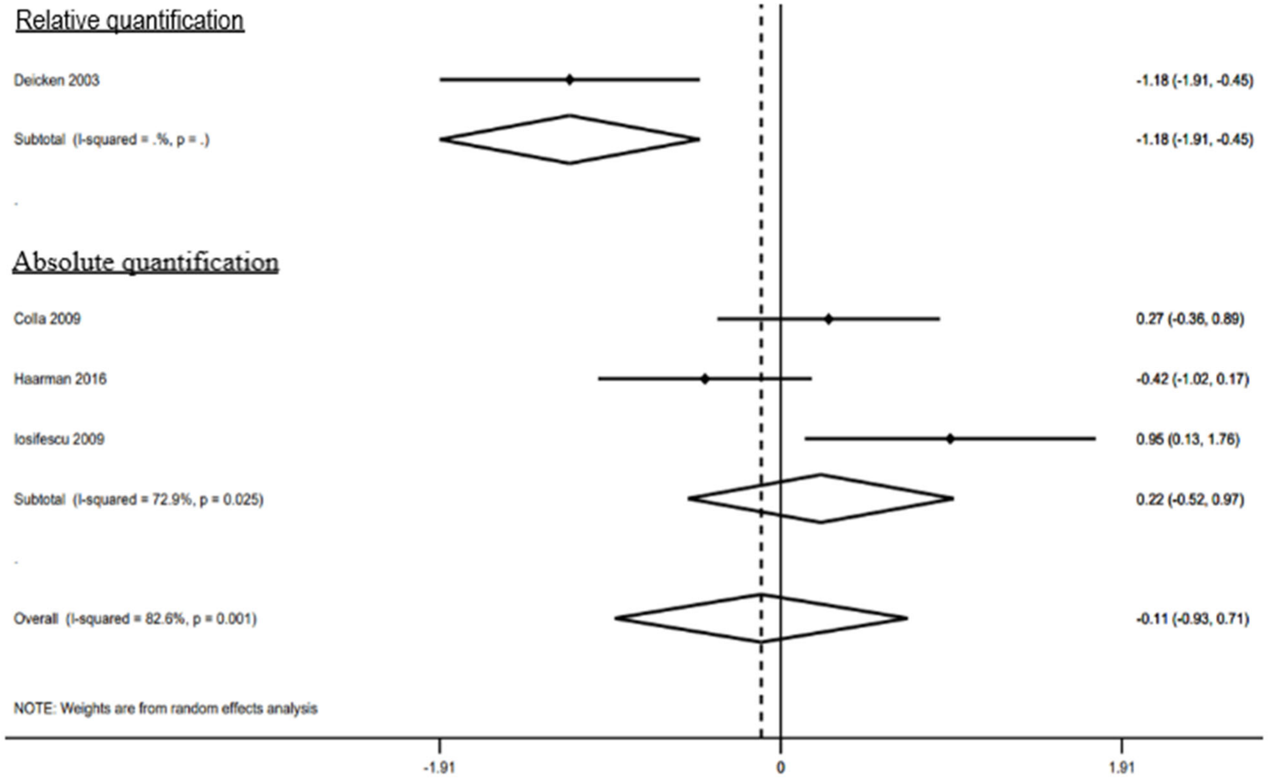

ACC:

Supplementary Figure S6A: Studies SMDs of glutamine differences between euthymic BD patients and controls in the ACC:

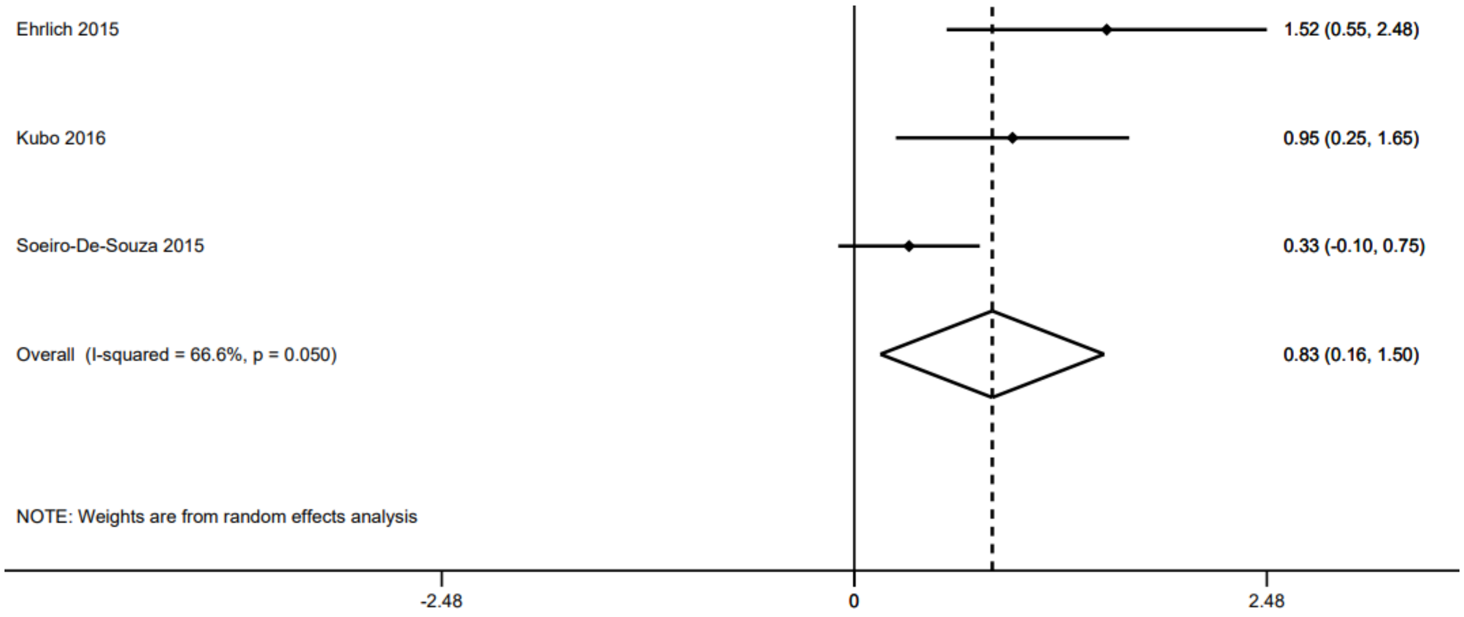

Supplementary Figure S6B: Studies SMDs of glutamine differences between euthymic BD patients and controls in the ACC according to quantification methodology:

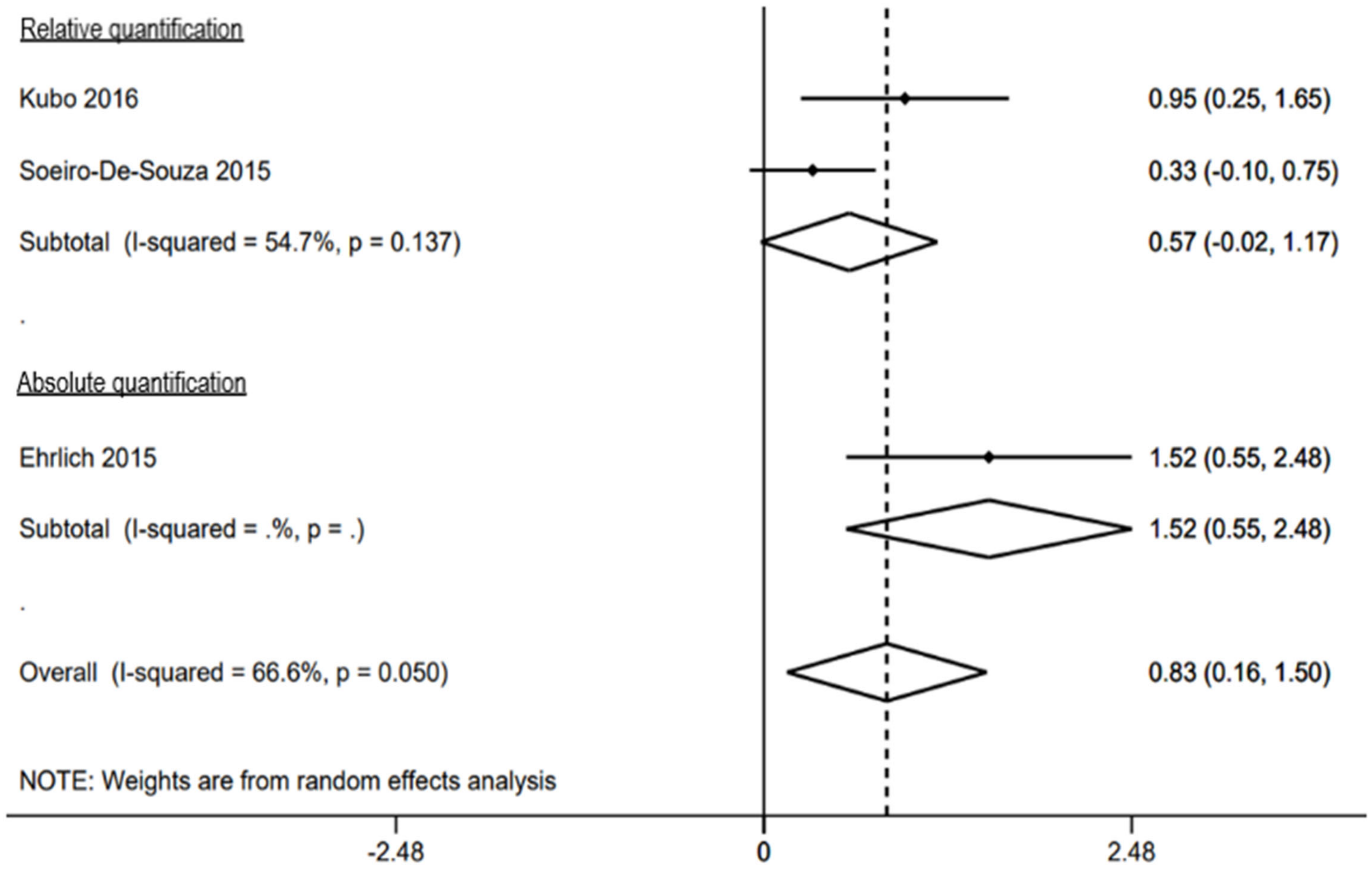

Supplementary Figure S7: Studies SMDs of glutamate differences between depressed BD patients and controls in the ACC:

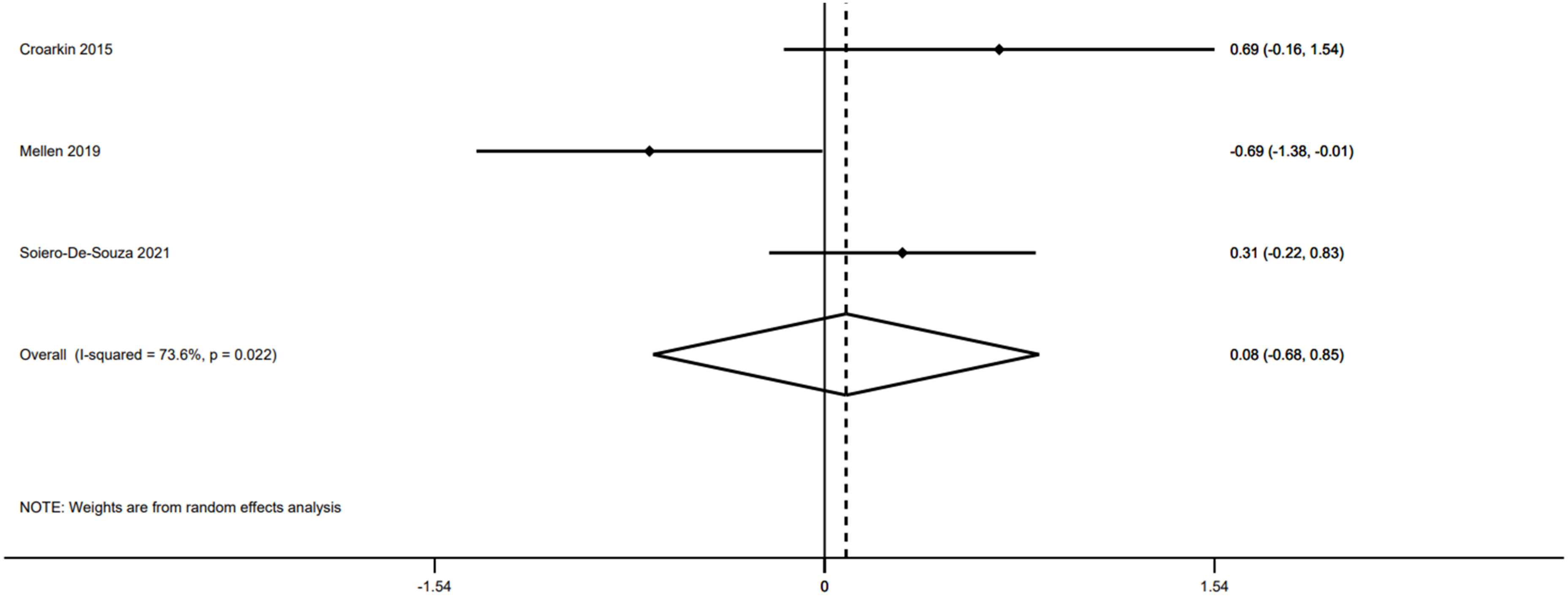

Supplementary Figure S8A: Studies SMDs of glutamate differences between euthymic BD patients and controls in the ACC:

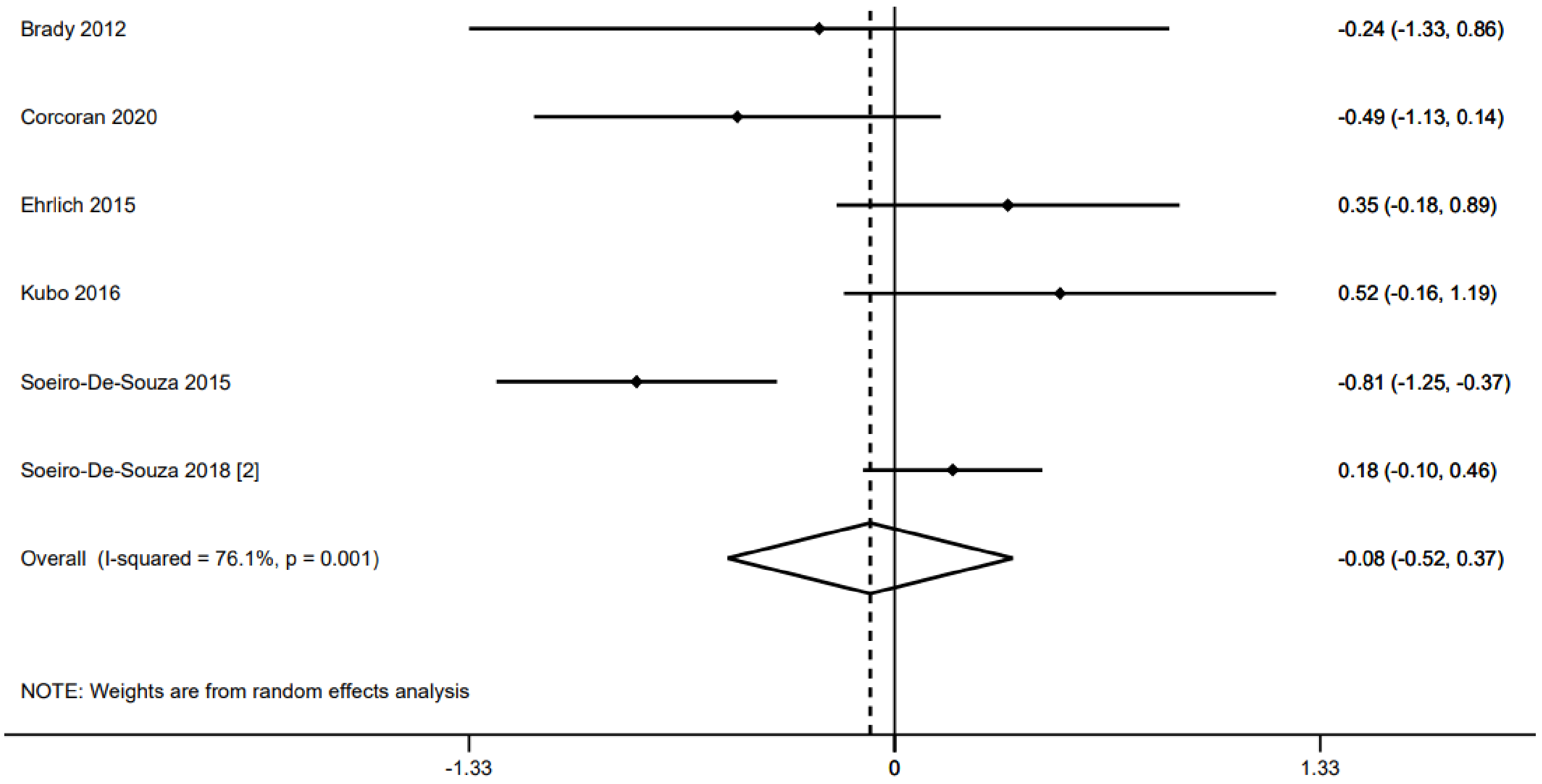

Supplementary Figure S8B: Studies SMDs of glutamate differences between euthymic BD patients and controls in the ACC according to quantification methodology:

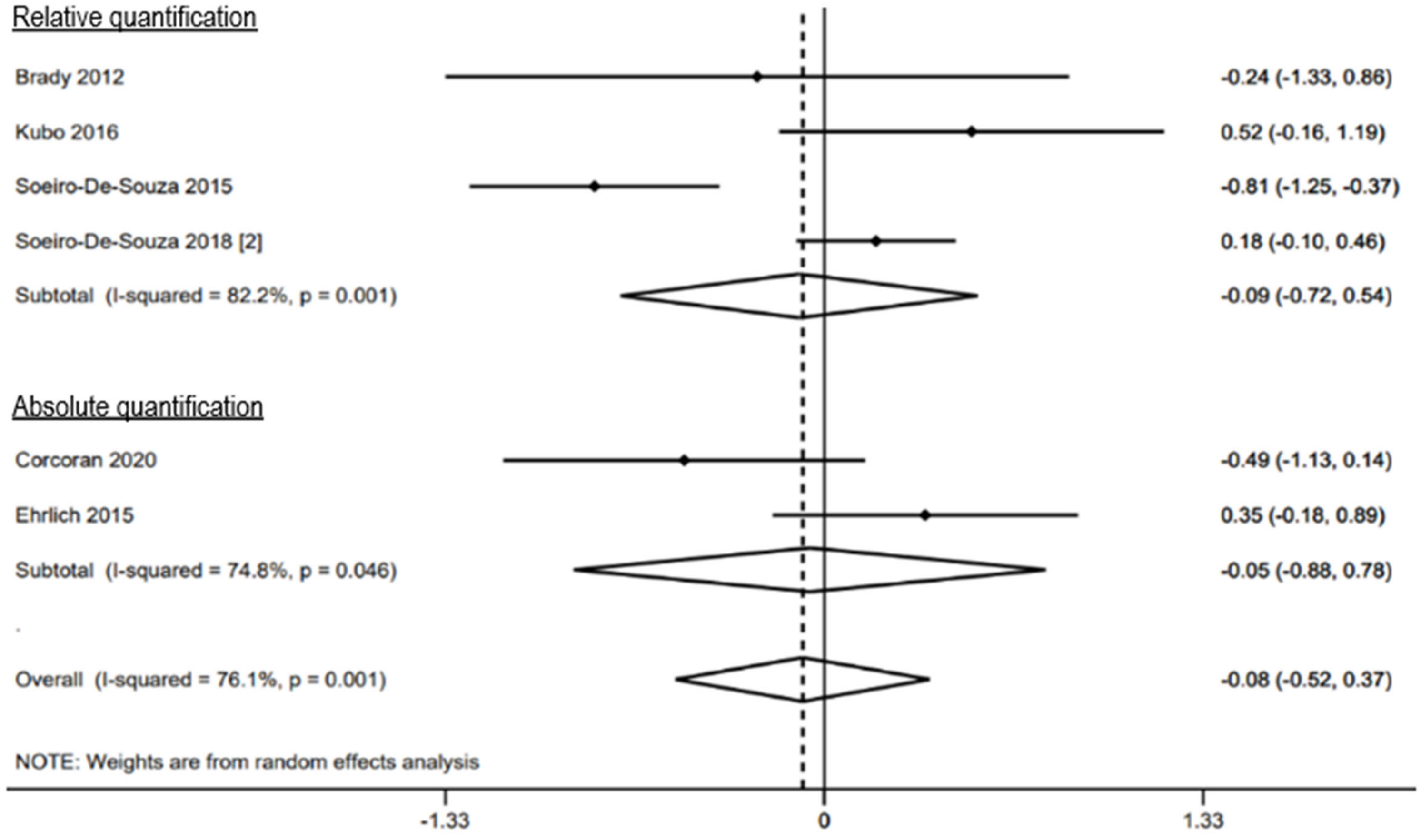

Supplementary Figure S8C: Studies SMDs of glutamate differences between euthymic BD patients and controls in the pregenual ACC and dorsal ACC:

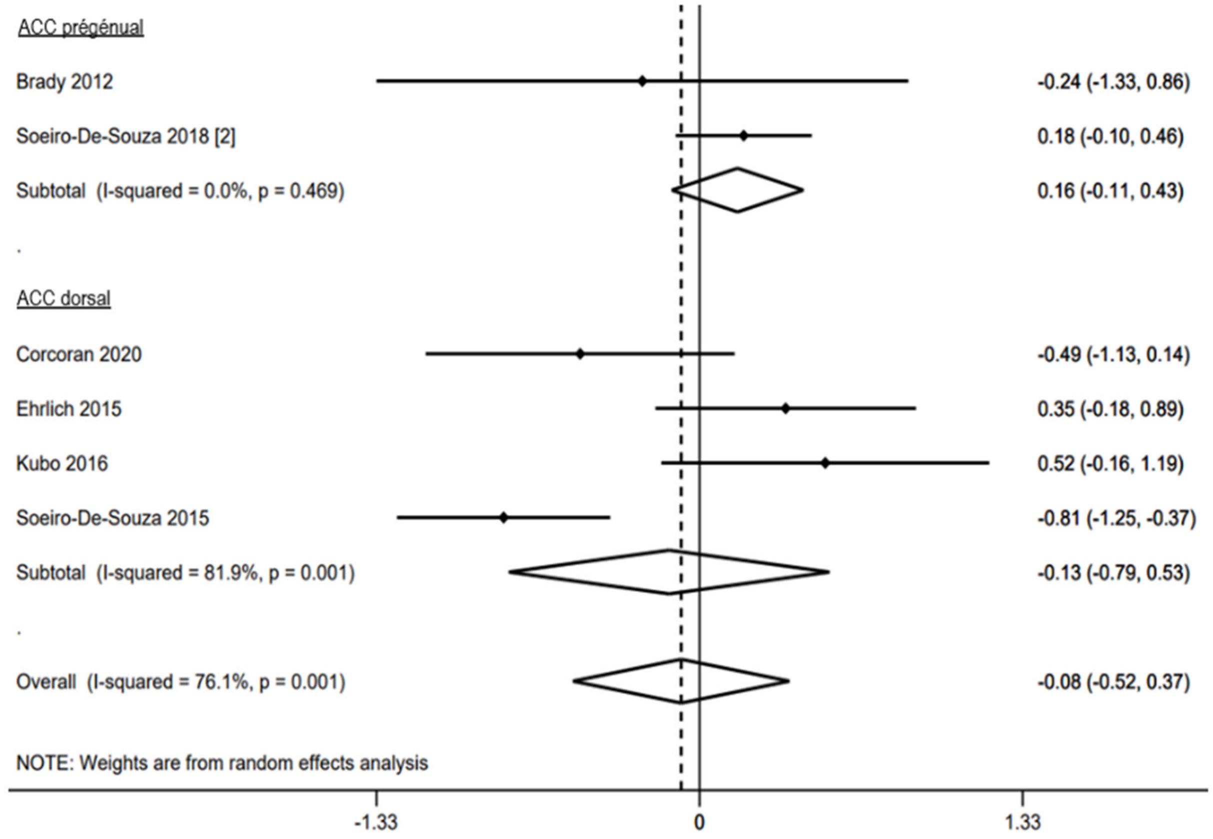

Supplementary Figure S9A: Studies SMDs of Glx differences between depressed BD patients and controls in the ACC:

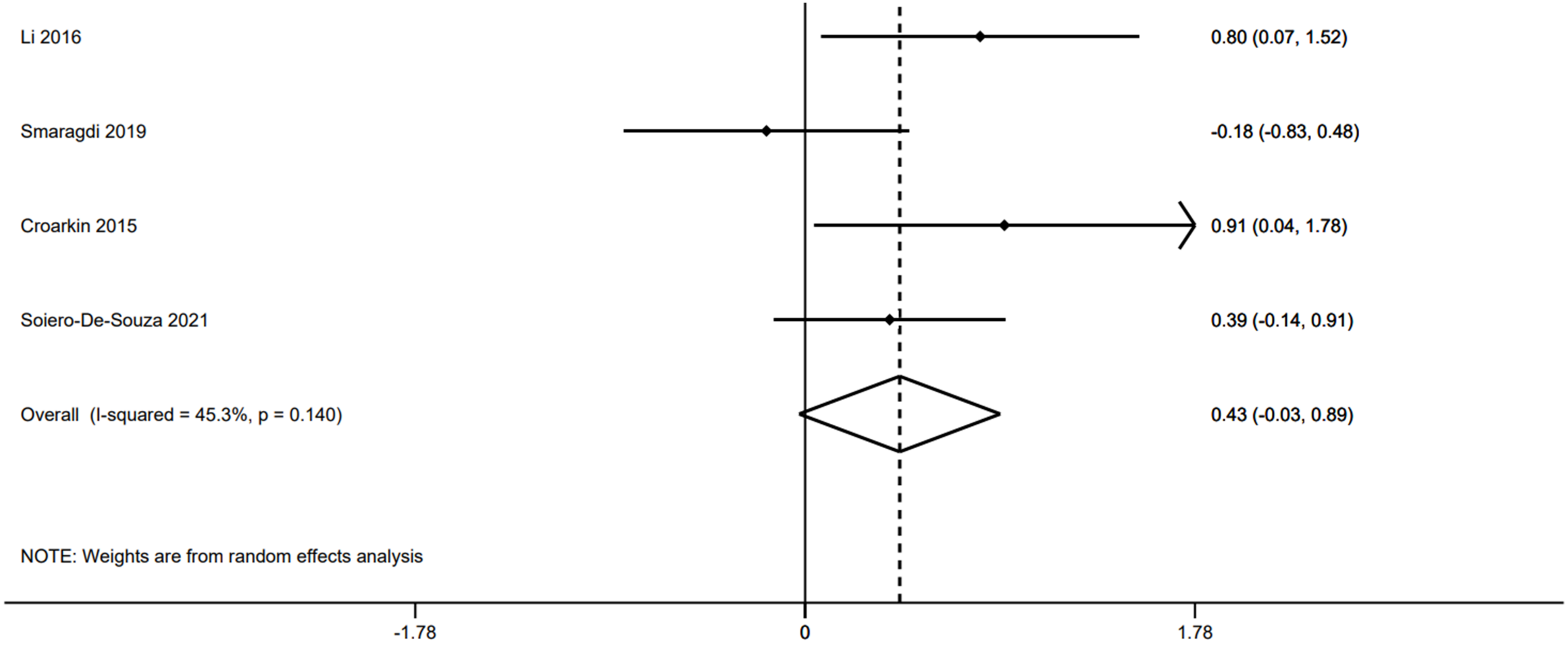

Supplementary Figure S9B: Studies SMDs of Glx differences between depressed BD patients and controls in the ACC according to quantification methodology:

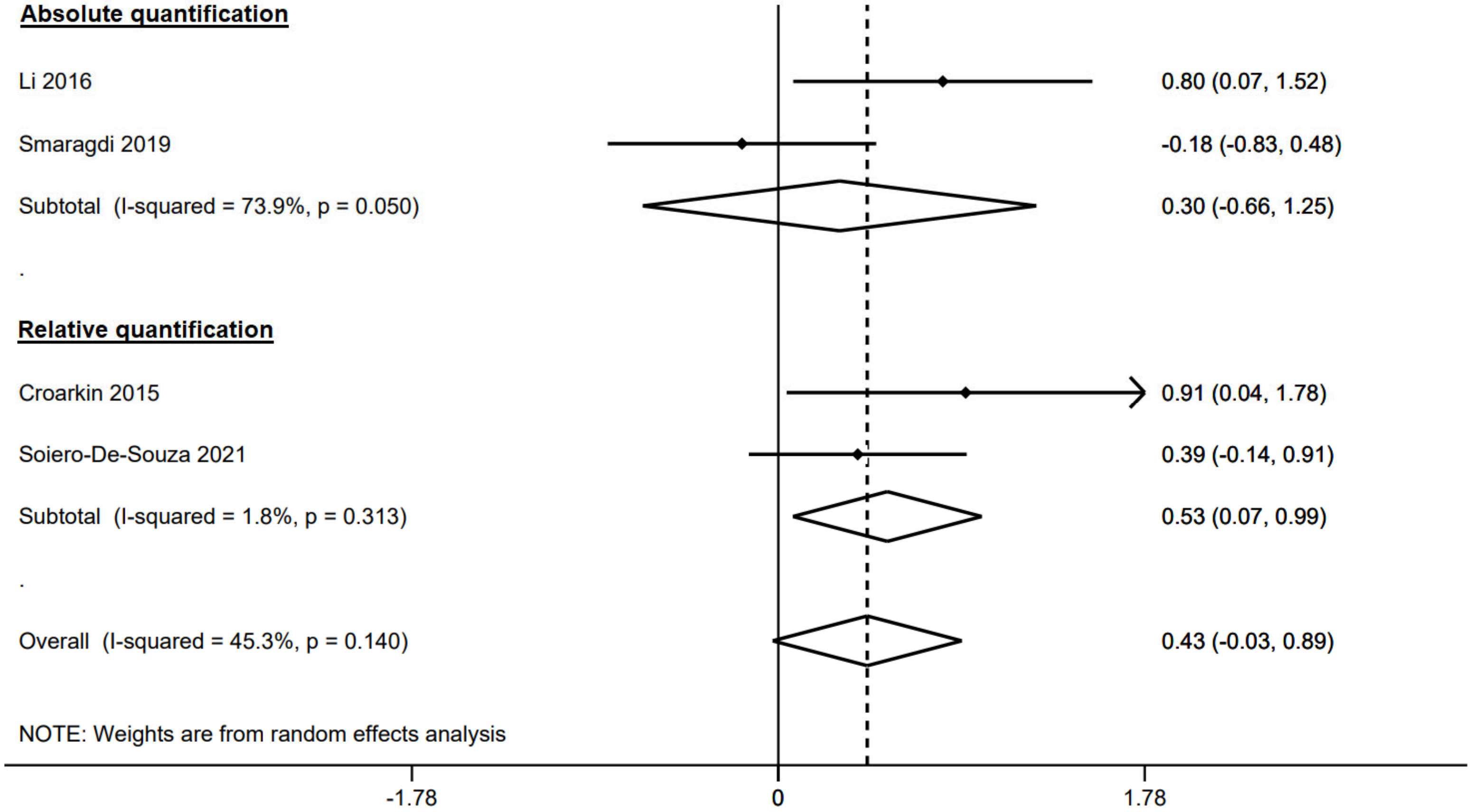

Supplementary Figure S10A: Studies SMDs of N-acetylaspartate differences between depressed BD patients and controls in the ACC:

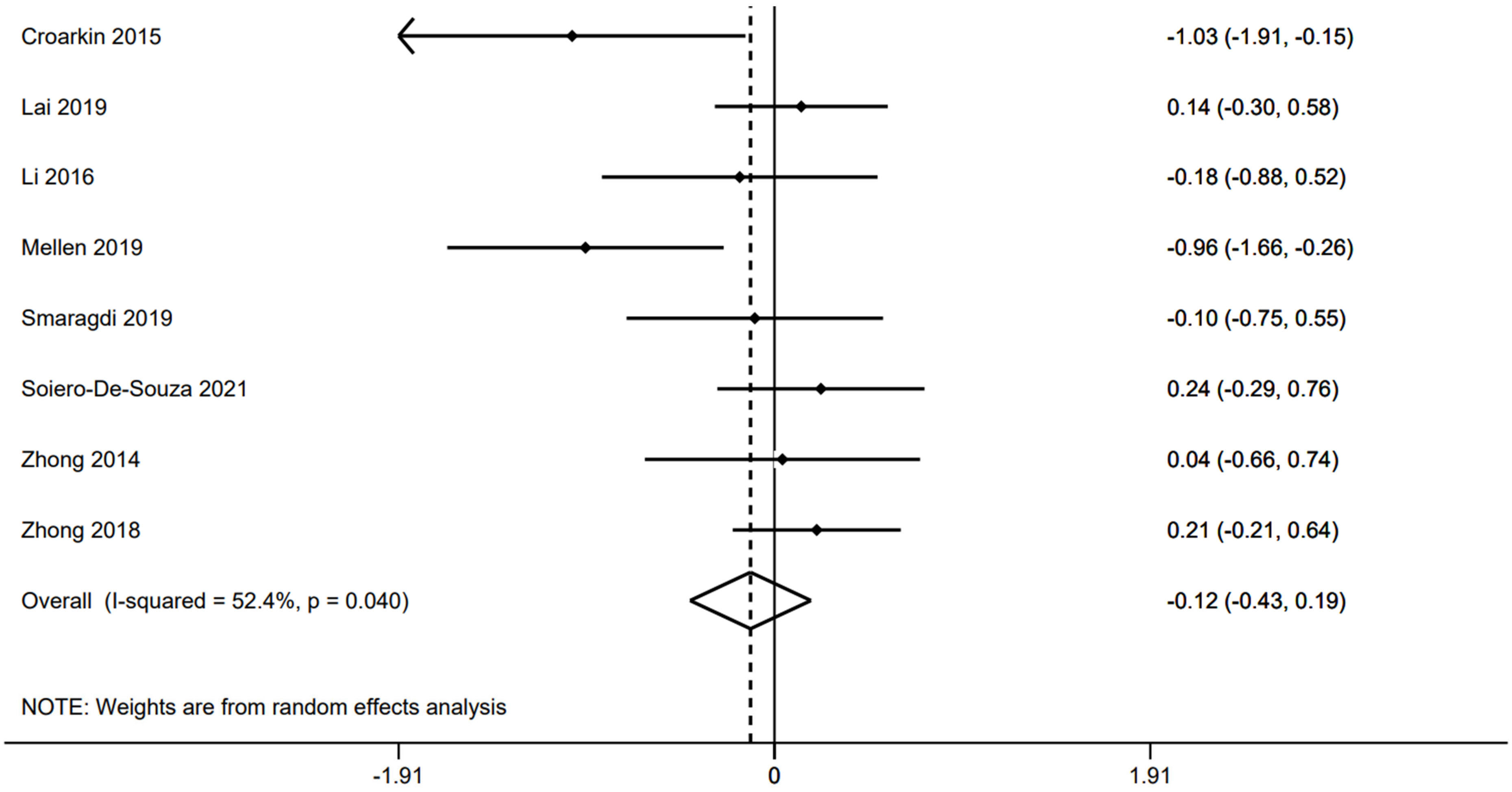

Supplementary Figure S10B: Studies SMDs of N-acetylaspartate differences between depressed BD patients and controls in the ACC according to quantification methodology:

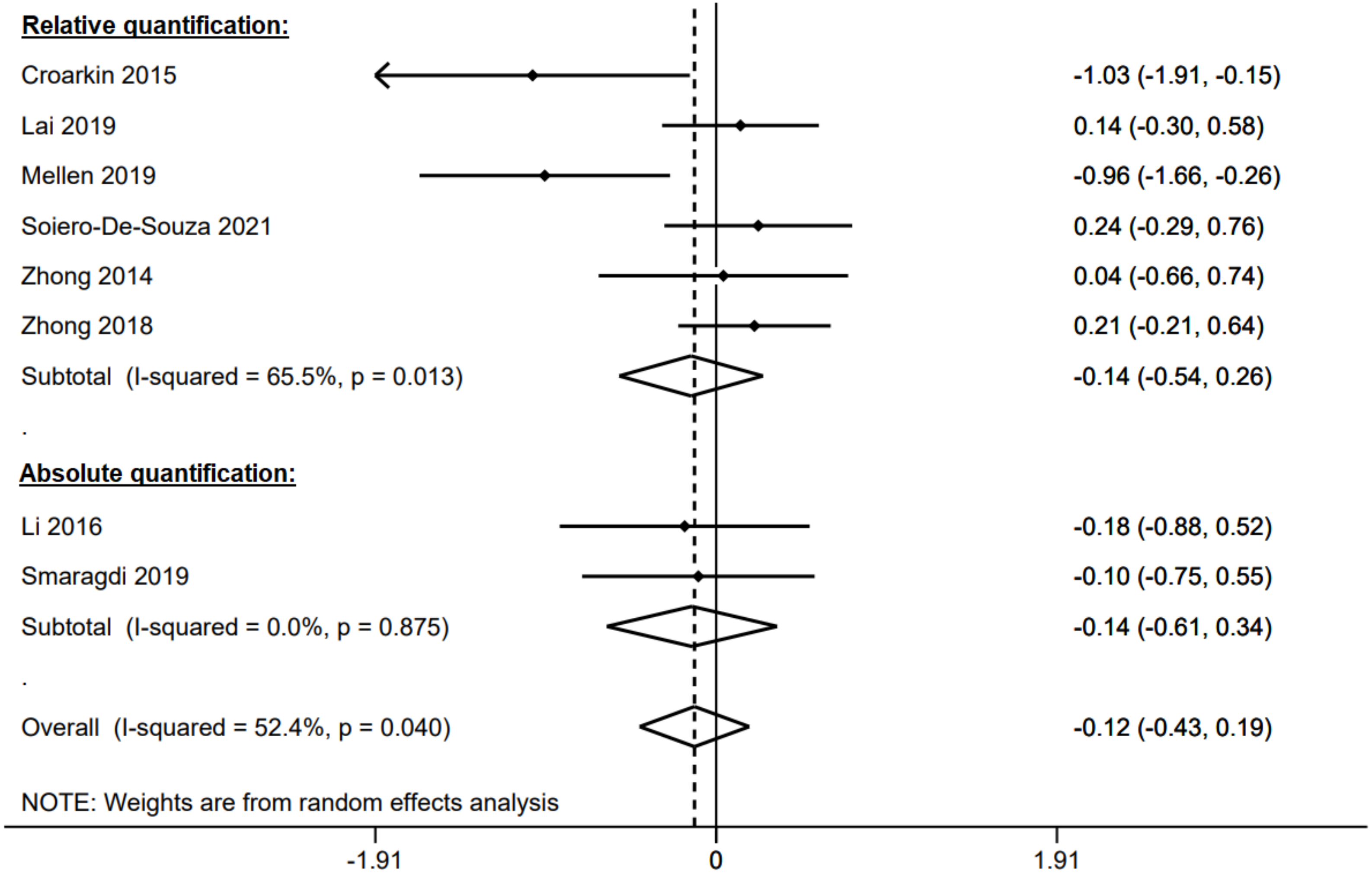

Supplementary Figure S10C: Studies SMDs of N-acetylaspartate differences between depressed BD patients and controls in the dorsal ACC:

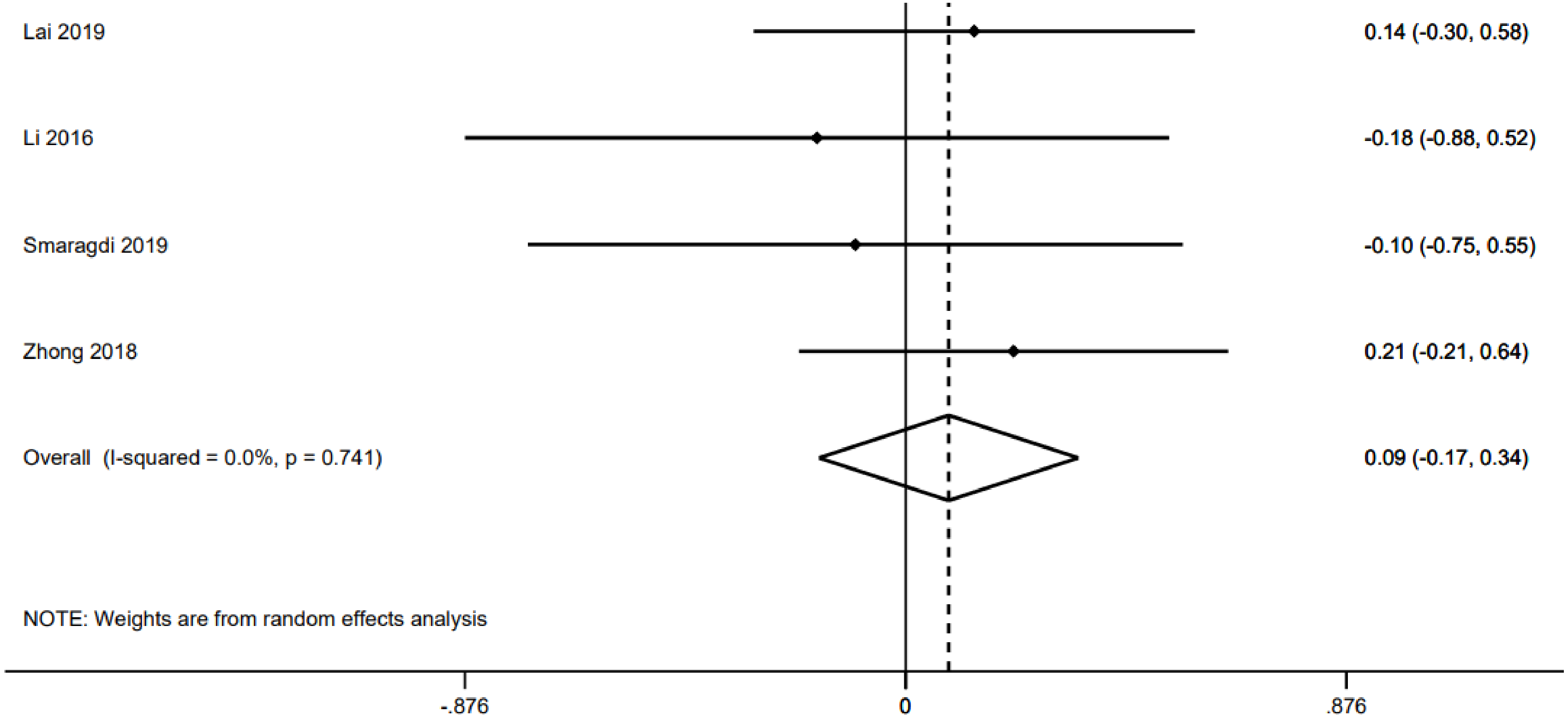

Supplementary Figure S10D: Studies SMDs of N-acetylaspartate differences between depressed BD patients and controls in the pregenual ACC:

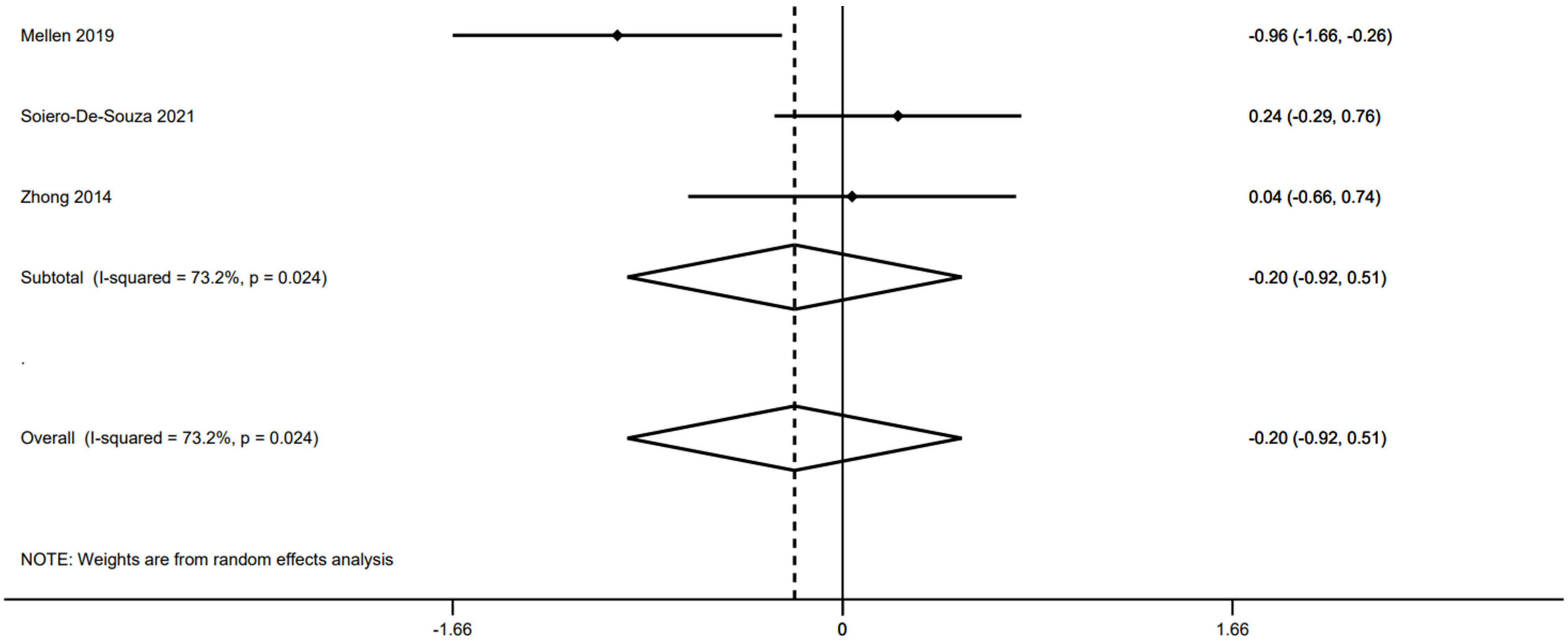

Supplementary Figure S11A: Studies SMDs of N-acetylaspartate differences between euthymic BD patients and controls in the ACC:

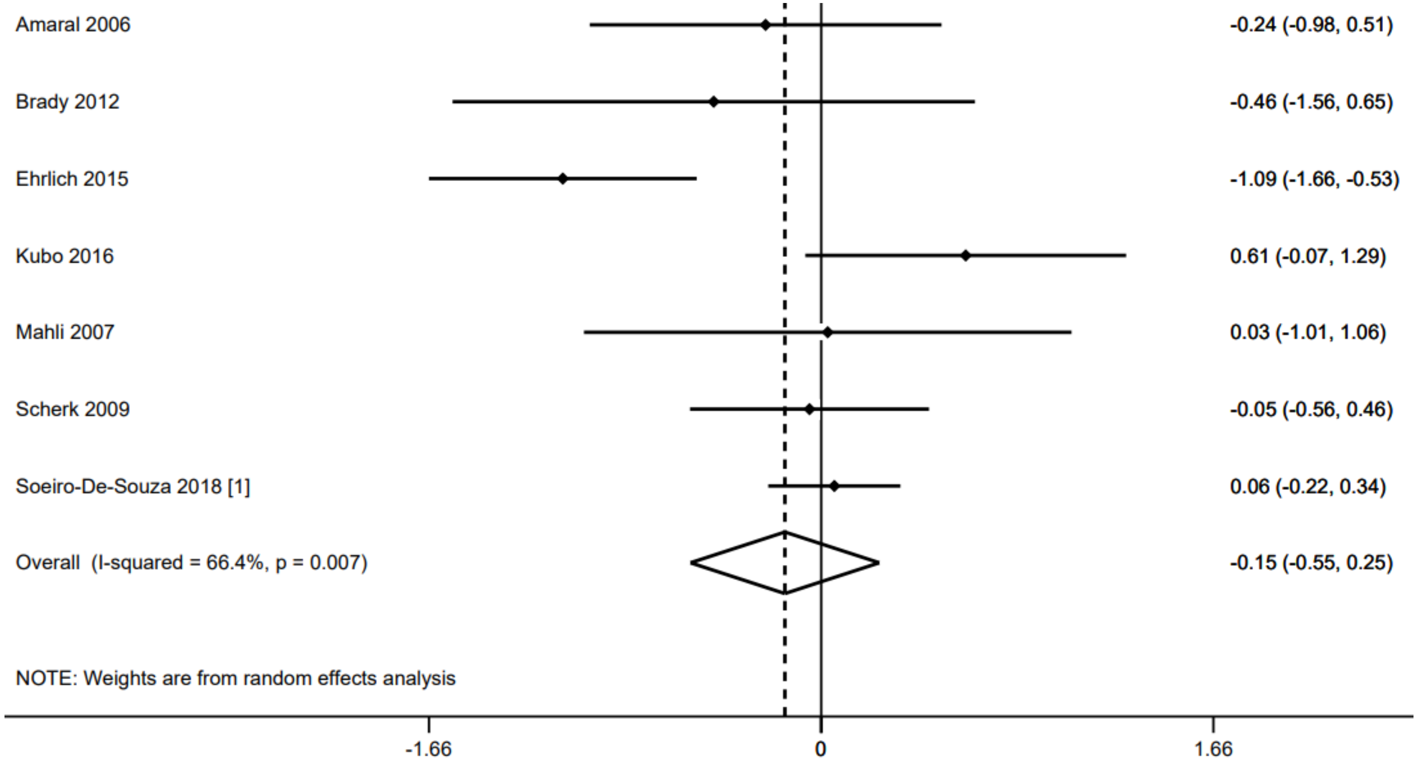

Supplementary Figure S11B: Studies SMDs of N-acetylaspartate differences between euthymic BD patients and controls in the ACC according to quantification methodology:

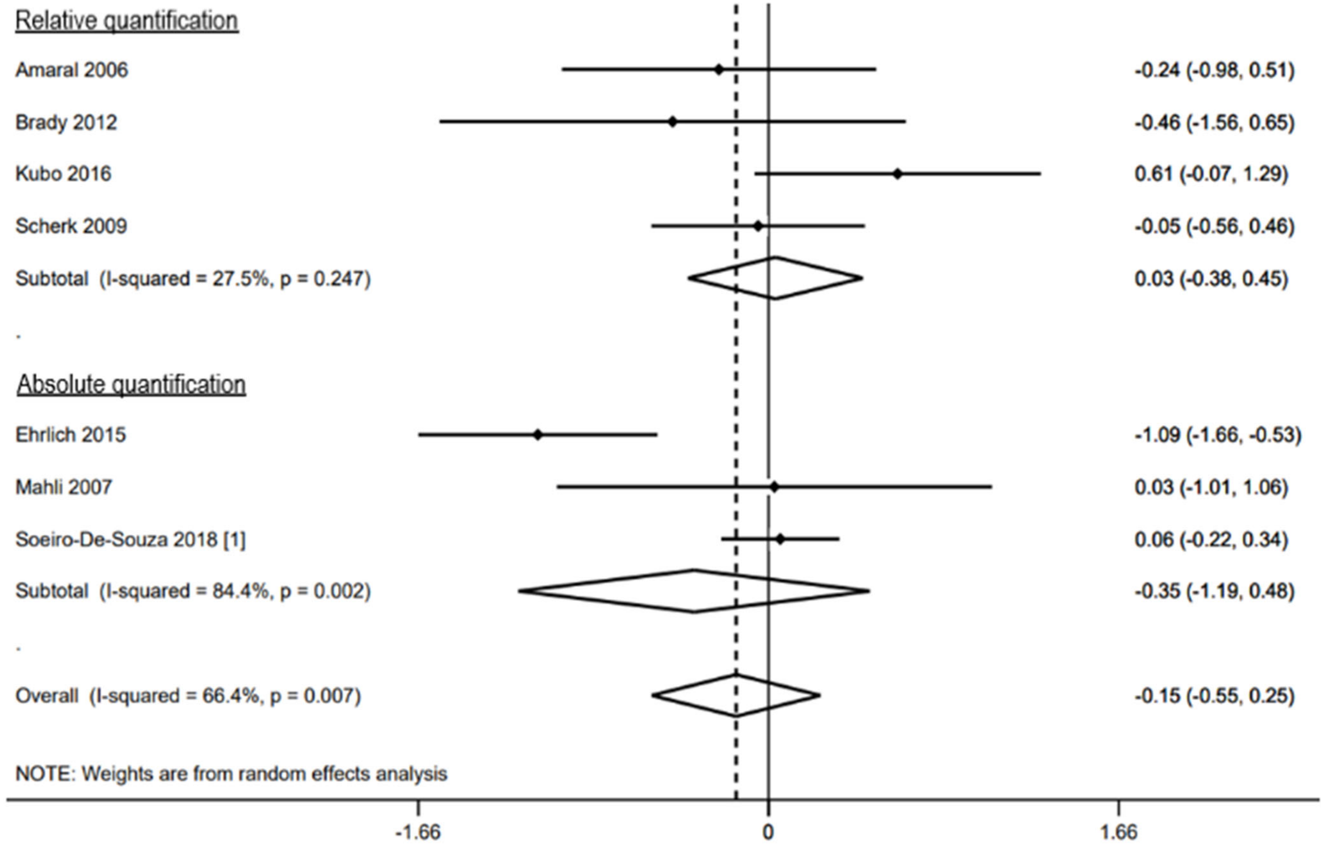

Supplementary Figure S11C: Studies SMDs of N-acetylaspartate differences between euthymic BD patients and controls in the dorsal ACC:

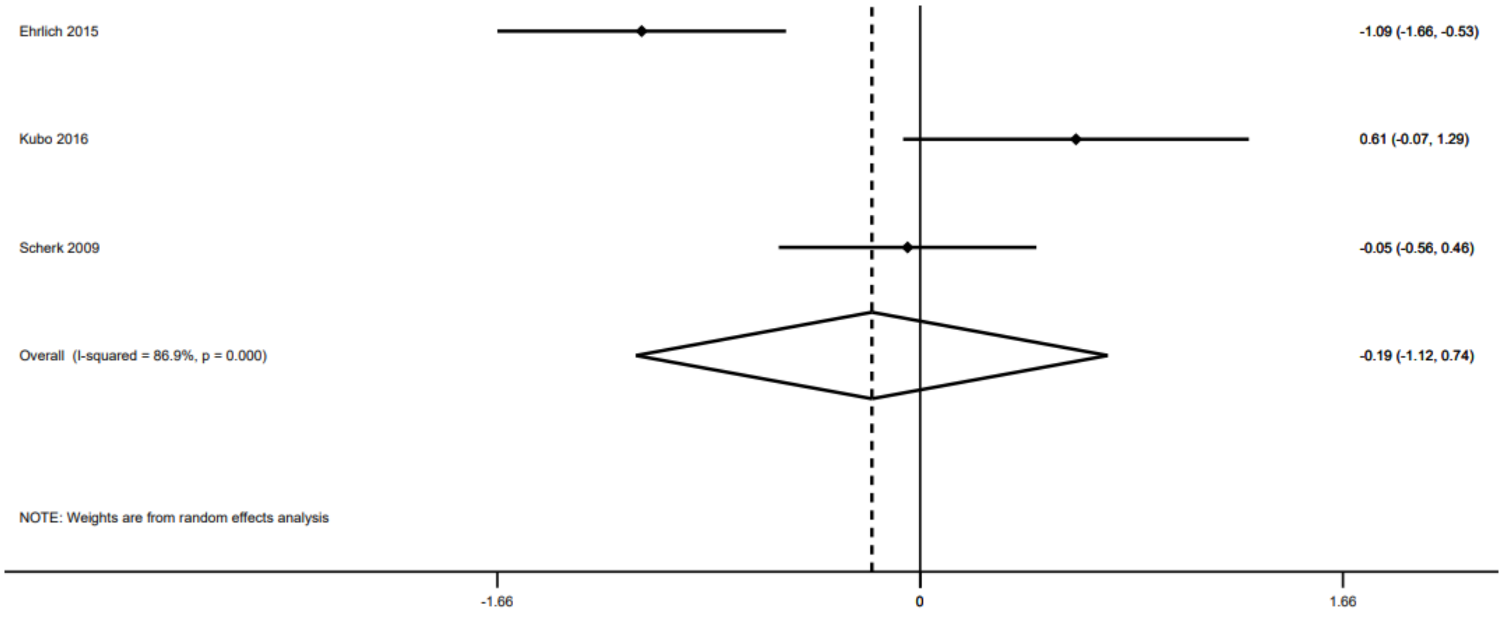

Supplementary Figure S11D: Studies SMDs of N-acetylaspartate differences between euthymic BD patients and controls in the pregenual ACC:

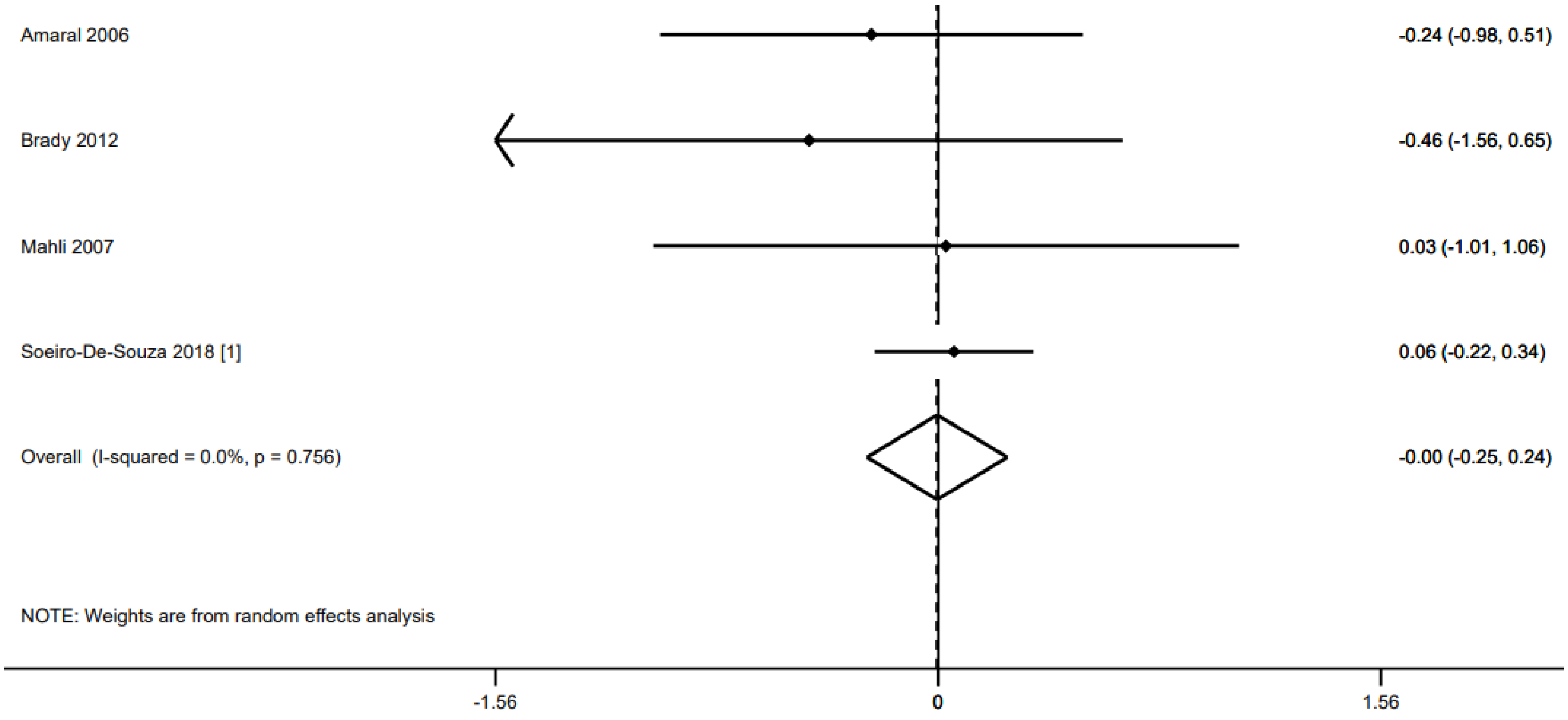

PFC:

Supplementary Figure S12: Studies SMDs of N-acetylaspartate differences between depressed BD patients and controls in the right wmPFC:

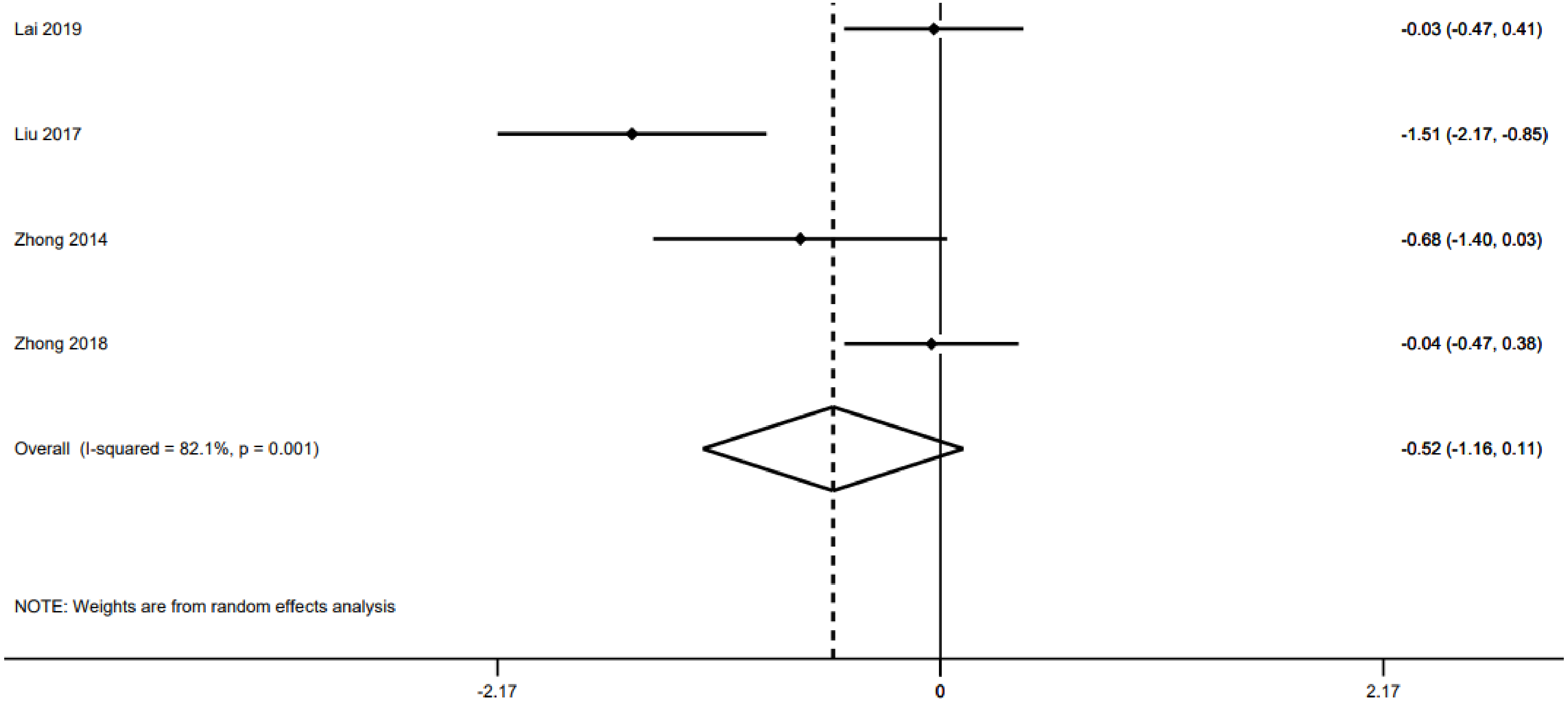

Supplementary Figure S13: Studies SMDs of N-acetylaspartate differences between depressed BD patients and controls in the left dlPFC:

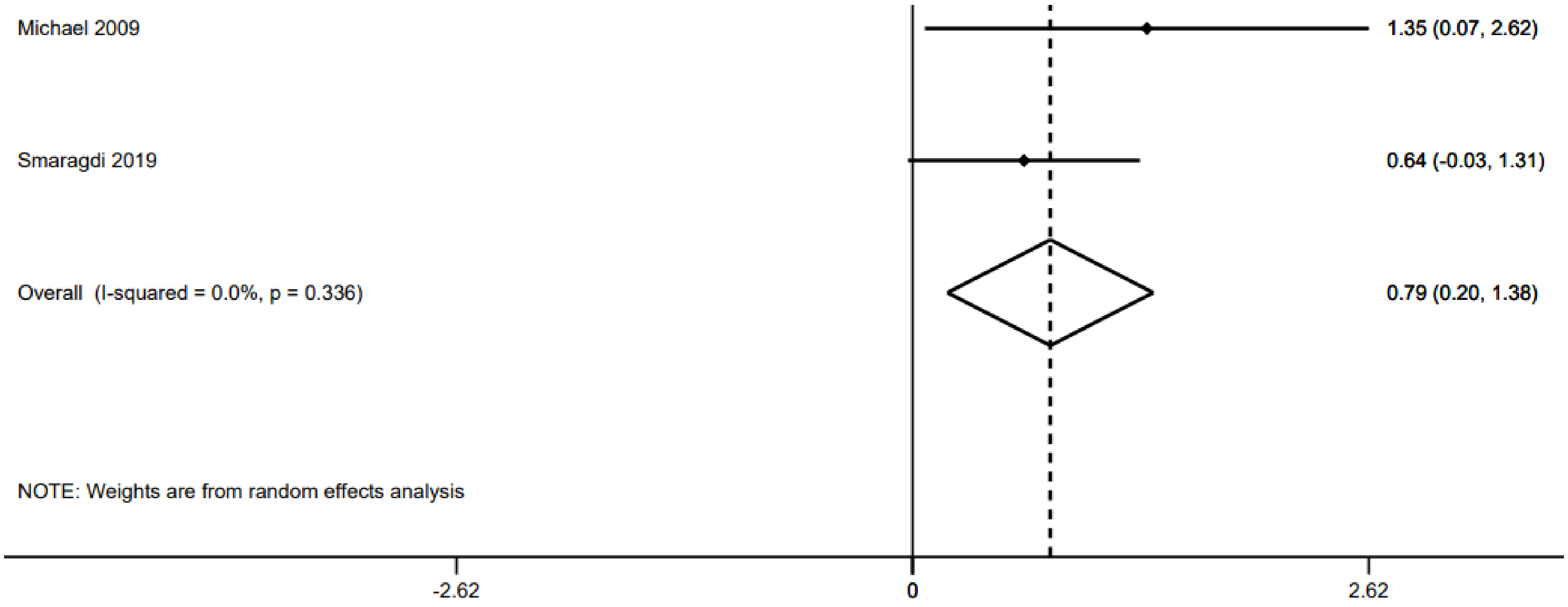

Supplementary Figure S14: Studies SMDs of N-acetylaspartate differences between euthymic BD patients and controls in the left dlPFC:

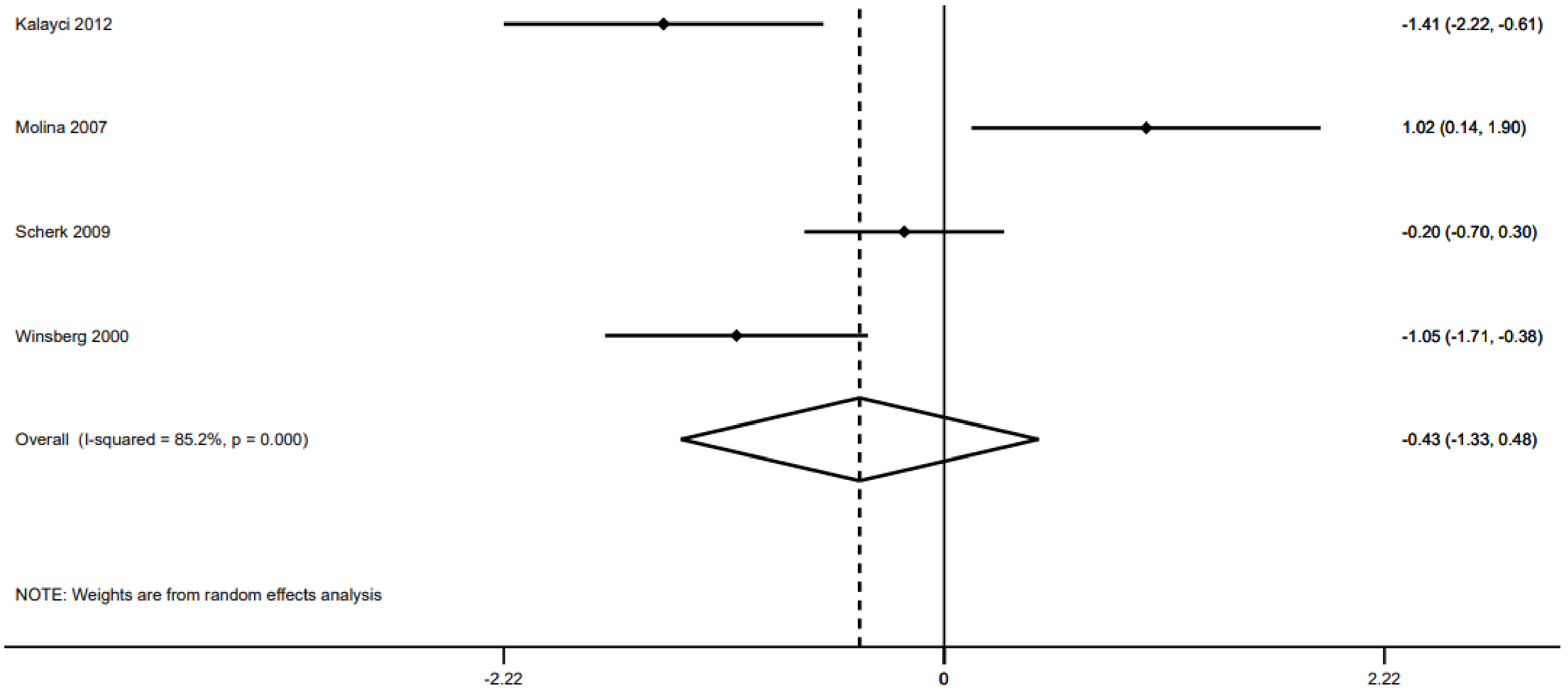

Supplementary Figure S15A: Studies SMDs of N-acetylaspartate differences between euthymic BD patients and controls in the right dlPFC:

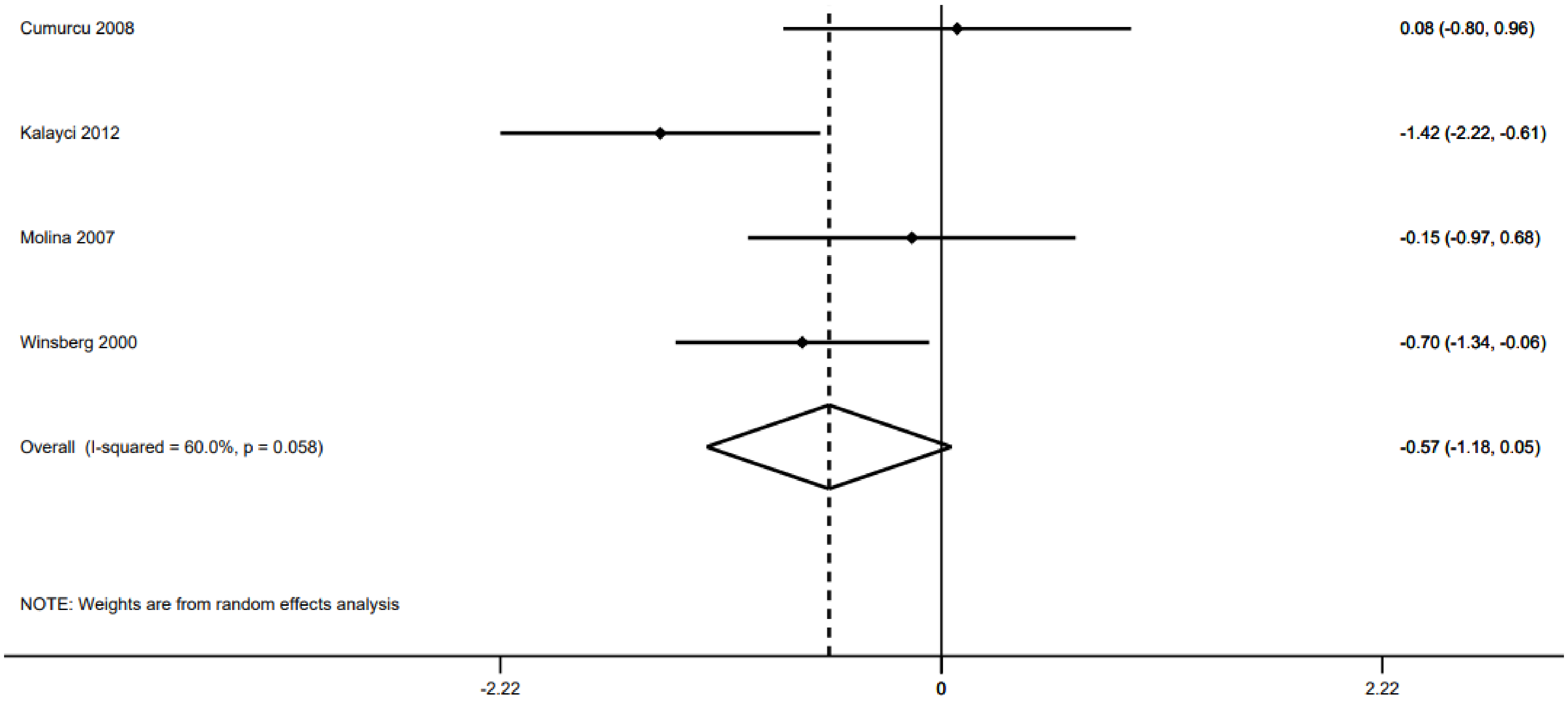

Supplementary Figure S15B: Studies SMDs of N-acetylaspartate differences between euthymic BD patients and controls in the right dlPFC according to the quantification methodology:

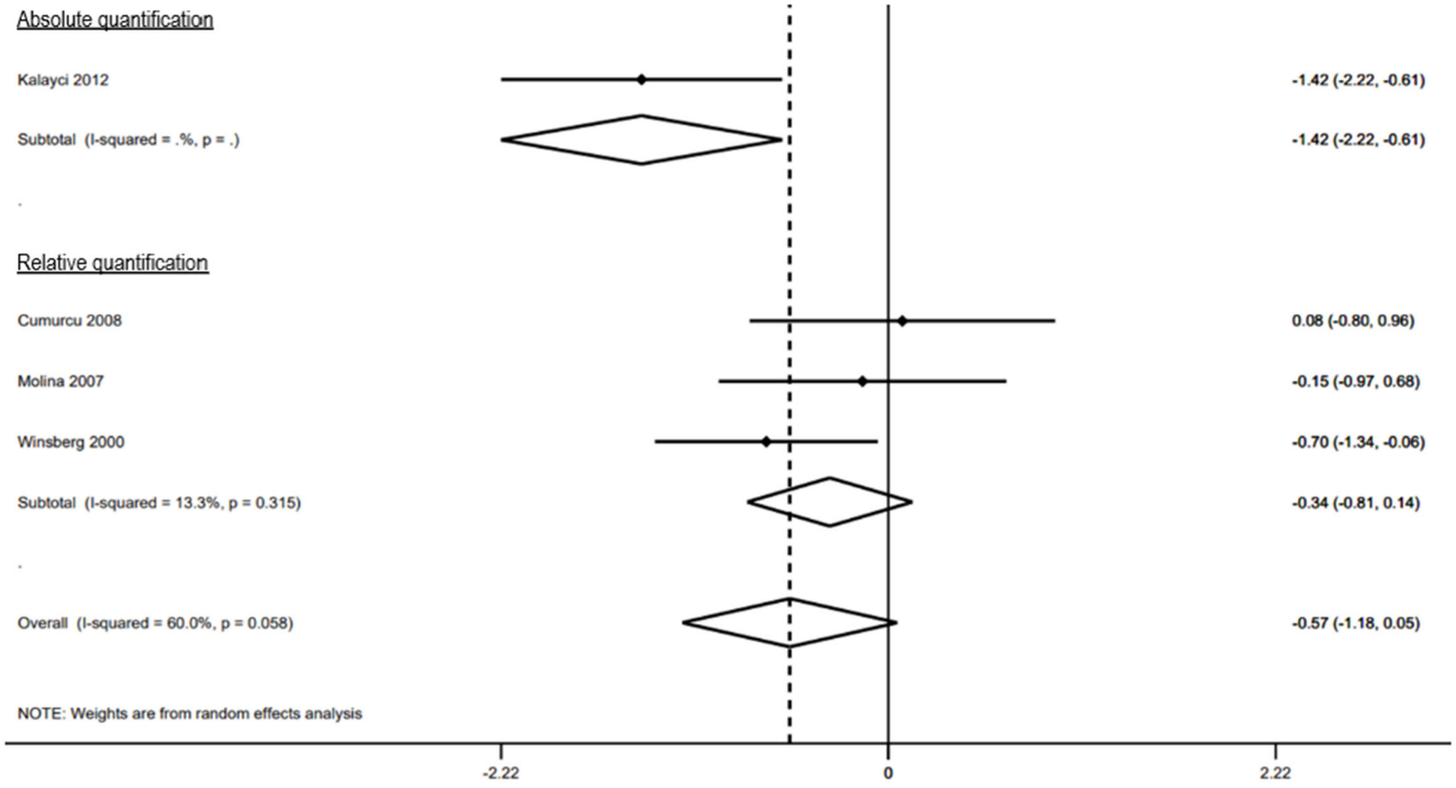

Supplementary Figure S16: Studies SMDs of Glx differences between depressed BD patients and controls in the left dlPFC:

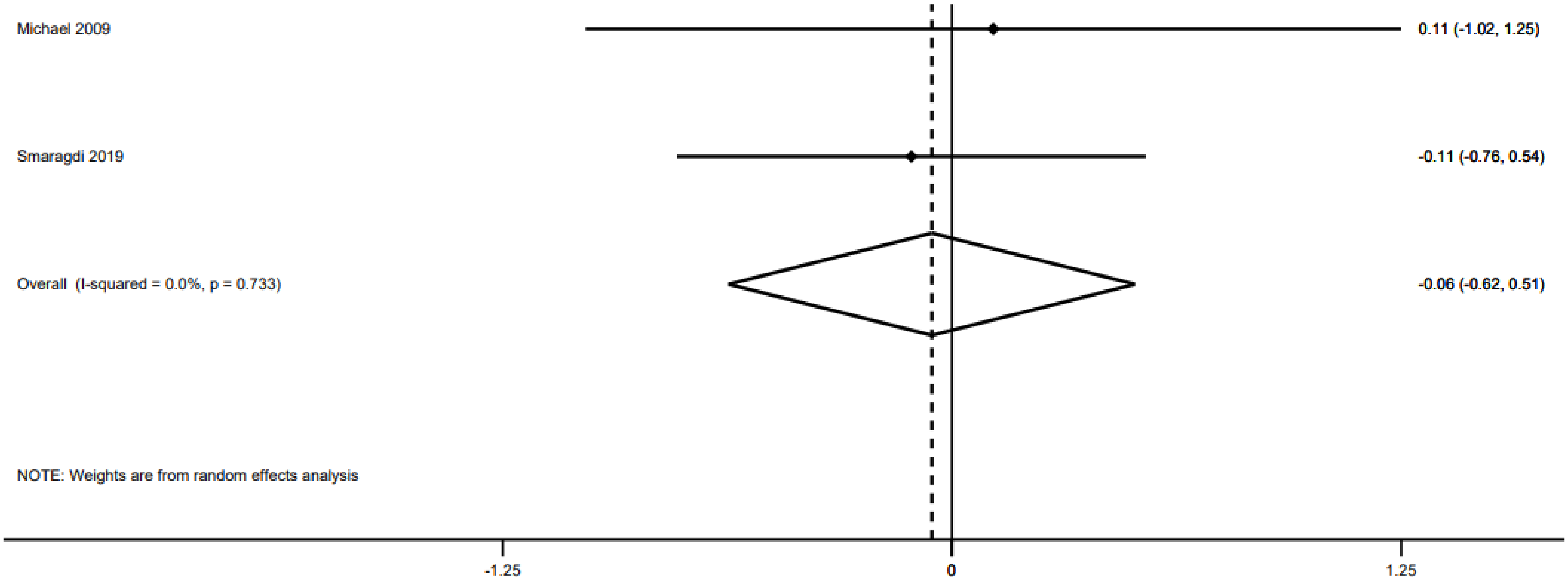

Supplement: Supplementary file 1 [file ijms-23-08974-s001.zip › ijms-1832409-supplementary.pdf]
